# Occurrence of *Eucoleus aerophilus* in wild and domestic animals: a systematic review and meta-analysis

**DOI:** 10.1186/s13071-023-05830-0

**Published:** 2023-07-20

**Authors:** Małgorzata Samorek-Pieróg, Tomasz Cencek, Emilia Łabuć, Małgorzata Pac-Sosińska, Mateusz Pieróg, Weronika Korpysa-Dzirba, Aneta Bełcik, Ewa Bilska-Zając, Jacek Karamon

**Affiliations:** 1 Department of Parasitology and Invasive Diseases, National Veterinary Research Institute, Partyzantów 57 Avenue, 24-100 Puławy, Poland; 2 Institute of Biological Sciences, Laboratory of Bioinformatics, University of Maria Curie-Skłodowska, Akademicka 19, 20-033 Lublin, Poland; 3 Institute of Biological Sciences, Department of Animal Physiology and Pharmacology, University of Maria Curie-Skłodowska, Akademicka 19, 20-033 Lublin, Poland

**Keywords:** *Eucoleus aerophilus*, *Capillaria aerophila*, Prevalence, Systematic review, Meta-analysis

## Abstract

**Background:**

*Eucoleus aerophilus* (syn. *Capillaria aerophila*) is a nematode with a worldwide geographical distribution. It causes a disease called lung capillariosis by affecting the respiratory tract of wild and domestic animals, and has also occasionally been described in humans. Despite steady increases in knowledge of the morphology of this neglected parasite, many aspects are still poorly understood. Epidemiological data regarding, for example, geographic distribution, range of hosts, clinical relevance and the actual zoonotic potential of this nematode are scarce and incomplete.

**Methods:**

This article is a systematic review based on the screening of three databases (PubMed, Web of Science and Science Direct) to identify eligible studies published from 1973 to the end of 2022.

**Results:**

From a total of 606 studies describing the occurrence of *E. aerophilus*, 141 articles from 38 countries worldwide were included in this meta-analysis, all of which presented results obtained mainly with flotation and necropsy. Due to the occurrence of *E. aerophilus* in many different species and different matrices (lungs and faeces), we decided to conduct the meta-analysis separately for each species with a given matrix. This systematic review confirmed the status of the Red fox as the main reservoir and main transmitter of *E. aerophilus* (average prevalence of 43% in faeces and 49% in lungs) and provided evidence of a higher prevalence of *E. aerophilus* in wild animals in comparison to domestic animals, such as dogs (3% in faeces) and cats (2% in faeces and 8% in lungs). Previous studies have investigated many host-related factors (age, sex, environmental/living conditions) in relation to the prevalence of *E. aerophilus*, but they show wide variations and no simple relationship has been demonstrates. Furthermore, mixed infections with other pulmonary nematodes, such as *Crenosoma vulpis* and/or *Angiostrongylus vasorum*, are reported very frequently, which greatly complicates the diagnosis.

**Conclusions:**

This systematic review focused on identifying data gaps and promoting future research directions in this area. To the best of our knowledge, this is the first systematic review that evaluates and summarizes existing knowledge on the occurrence and prevalence of *E. aerophilus* in wild and domestic animals originating from different geographical locations worldwide.

**Graphical Abstract:**

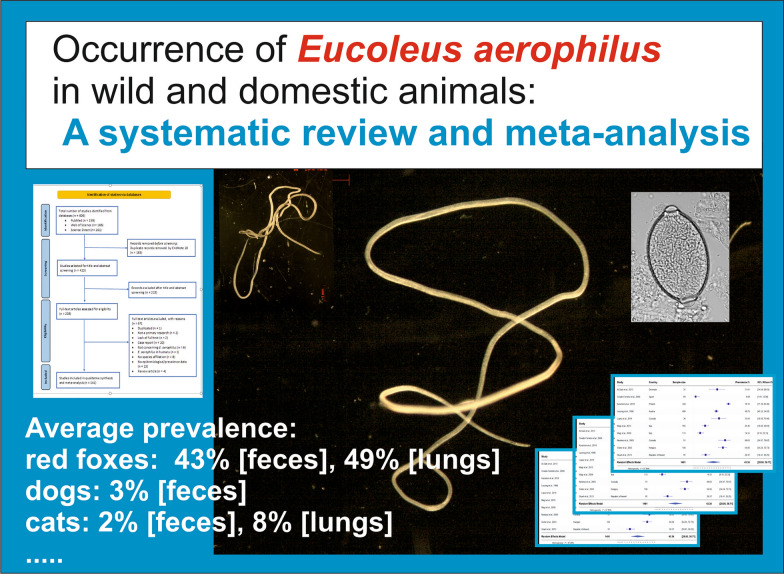

**Supplementary Information:**

The online version contains supplementary material available at 10.1186/s13071-023-05830-0.

## Background

*Eucoleus aerophilus* (Creplin, 1839) Dujardin, 1845 (syn. *Capillaria aerophila*) is a nematode with a worldwide geographical distribution. It belongs to the order Trichocephalida and family Capillariidae (Railiet, 1915) [1]. *Eucoleus aerophilus* causes a disease called lung capillariosis by affecting the respiratory tract of wild carnivores and insectivores (mainly foxes, coyotes, wolves) and domestic animals (dogs, cats) [2]; also, it is potentially a zoonotic parasite and has occasionally been described in humans [3, 4]. It is not fully determined whether this parasite has a direct or indirect life-cycle, and there is some speculation that earthworms may act as intermediate hosts or paratenic hosts [5, 6]. In the review by Anderson [7], it was stated that Christenson [8] failed to experimentally infect cats and foxes by feeding them with larvated eggs; in contrast, Borovkova [7] was able to infect cats, dogs and foxes by feeding them with earthworms exposed to larvated eggs. Both studies were compromised by the likelihood of co-infections with *Eucoleus boehmi* in the canids they worked on. Many gaps in our knowledge of the biology of *E. aerophilus* remain today, and so far there are no studies confirming the role of the earlier mentioned invertebrates in the biology of this parasite [2, 5–7]. Adult worms live beneath the epithelium of the bronchioles, bronchi and trachea of the infected host, where they subsequently reproduce. Mature males reach 10–25 mm in length, while females reach 16–42 mm in length [9]. Mature females produce non-larvated eggs, which are coughed up and swallowed by the host, ultimately reaching the environment through the faeces. Eggs of *E. aerophilus* measure 60–83 µm × 25–40 µm, are barrel-shaped and have asymmetrically arranged bipolar plugs and walls with a network of anastomosing ridges and bridges [2, 10–12]. Released eggs embryonate within 5–6 weeks and remain viable for up to 1 year. The eggs can also mature within earthworms [5, 6]. Animals acquire infection through incidental ingestion of the larvated eggs. In the digestive tract of carnivores, the larvae hatch and within 7–10 days penetrate the intestinal wall and then, via the bloodstream or lymphatic vessels, reach the lungs, where they mature sexually (approximately 3–4 weeks after infection) [11].

In animals, infection with *E. aerophilus* can be either subclinical or lead to respiratory distress that ranges from mild disease to severe and potentially fatal pneumonia. The lung parenchyma becomes damaged by adult parasites, which is the causal factor resulting in bronchovesicular breath sounds, sneezing, wheezing and chronic dry or moist productive cough, particularly when the infection is accompanied by secondary bacterial infections [13]. Heavy infection can lead to life-threatening bronchopneumonia and respiratory failure [11].

Despite increases in our knowledge of the morphology of this neglected parasite [9, 11, 14–16], many aspects are still poorly understood. Epidemiological data regarding, for example, geographic distribution, range of hosts, clinical relevance and the actual zoonotic potential of this nematode are scarce and incomplete [9, 11]. Among wild animals, foxes are believed to be the most common host and reservoir of *E. aerophilus*, the prevalence of which is usually high, such as, for example, 41.8% in Italy [17], 46.8% in the Netherlands [18], 66% in Hungary [19], 74.1% in Denmark [20] and 88% in Norway [21]. Nevertheless, the spread of this parasite has been observed in companion animals (dogs and cats) in many parts of the world over the past few years, including in Italy [22, 23], Germany [24], Poland [25], Hungary [26], Romania [27], Canada [28], India [29, 30] and USA [31–33], among others. Moreover, genetic research has confirmed that some sub-populations of *E. aerophilus* co-infect wild and domestic animals [5]. The increase in the Red fox population in the last two decades [34–37], the decline in natural habitats due to progressive urbanization and the increased access of humans and companion animals to wilderness areas play a crucial role in the spread of this lungworm and the infection of companion animals [2, 5, 13]. This phenomenon significantly increases the risk of transmission of *E. aerophilus* to humans. Cases of pulmonary capillariosis in humans are described in the literature [3, 4, 38, 39], most of which were diagnosed incidentally as the clinical symptoms of the disease are usually non-specific or resemble those of bronchial pneumonia or even lung cancer. These incidental diagnoses suggest a possible underestimation of data on the prevalence of *E. aerophilus* in humans.

*Eucoleus aerophilus* as a nematode that parasitizes the lungs of companion animals is still an underestimated problem among veterinarians, possibly due to the lack of basic parasitological research conducted in this direction. Specific coprological diagnosis of *E. aerophilus* can be challenging because of the similarity in the structure of the eggs with those of other species infecting carnivores, such as the nasal parasite *E. boehmi* or the whipworm *Trichuris vulpis* [6, 23, 40]. Inaccurate diagnosis often results in a prolonged treatment of animals [11]. In Europe, the reported infection rate of *E. aerophilus* in foxes varies greatly, which may be the result of using different detection methods, such as examination of lung specimens and microscopic or PCR methods, which differ in sensitivity and specificity. From an epidemiological point of view, research into the occurrence and spread of *E. aerophilus* is very important, as it is a potential source of human infection.

The aim of this systematic review was to evaluate and summarize existing knowledge on the occurrence and prevalence of *E. aerophilus* in wild and domestic animals originating from different geographical locations worldwide. The information obtained was used to compile tables on the prevalence of this nematode or to emphasize the lack of reliable reports. In this review, we specified information on the methods and techniques used for the detection of *E. aerophilus* in different hosts and data on the intensity of infection and co-infections when available. The secondary aim of this systematic review was to estimate *E. aerophilus* occurrence and prevalence in different hosts worldwide to identify data gaps.

## Methods

This systematic review followed the Preferred Reporting Items For Systematic Reviews and Meta-Analysis (PRISMA) statement [41] and Meta-Analysis of Observational Studies in Epidemiology (MOOSE) consensus statement [42].

### Literature searches

Bibliographic searches of published studies were conducted on 20 February 2022 and again on 21 December 2022 to identify articles that had been published since the initial search. Three databases, namely PubMed (https://pubmed.ncbi.nlm.nih.gov), Web of Science (www.webofknowledge.com) and ScienceDirect (https://www.sciencedirect.com), were screened for studies using the following keywords and Boolean operator: “*Eucoleus aerophilus*” OR “*Capillaria aerophila*”. The results of these searches were combined and screened for duplicates using the EndNote 20 reference management tool (Clarivate, Philadelphia, PA, USA), and all duplicated articles were removed.

### Study selection criteria

**Fig. 1:**
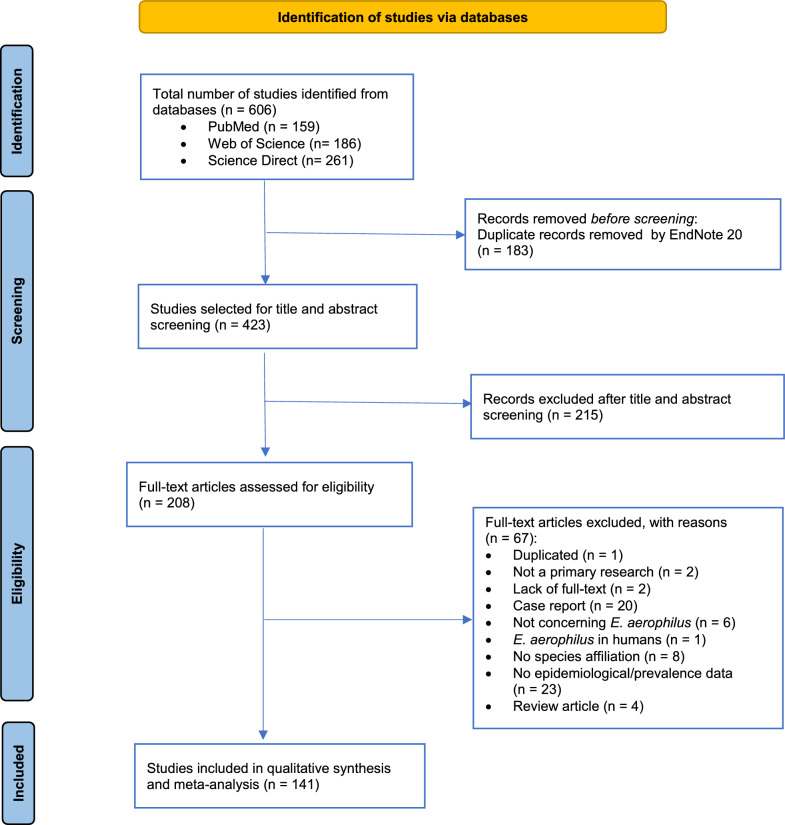
Flow diagram representing the search algorithm for *Ecuoleus aerophilus* studies in databases and the study selection process

The pre-selection of studies was made on the basis of the information contained in the title and abstract; if no decision could be made, the full text was checked. Next, full-text articles were assessed for eligibility according to the pre-determined inclusion/exclusion criteria. The inclusion criteria applied to select articles were: (i) cross-sectional or cohort studies; (ii) original peer-reviewed studies; (iii) studies containing extractable information on the prevalence of *E. aerophilus* in wild or domestic animals; (iv) studies providing a sufficient description of the method used; (v) studies providing an adequate description of the sample type; and (vi) available full-text articles. The articles considered not to be eligible for inclusion were those providing data on the occurrence of *E. aerophilus* in humans, case reports, reviews, book sections, retrospective studies, articles with no access to the full text, articles with no species affiliation to *E. aerophilus*, articles with no epidemiological/prevalence data on *E. aerophilus* and articles with no data on *E. aerophilus*. The study selection process is presented as a the flow chart in Fig. 1.

### Data extraction

Full texts of articles were checked, and essential data were extracted independently by two researchers (MSP and JK). In case of any doubts, the decision was made after discussion, and any disagreements were resolved by consensus. Information including the first author’s name, title of article, year of publication, type of study, time when the study was conducted, geographic location, animal species tested, animal age, animal sex, sample size, sample type, sampling strategy, stages of detected *E. aerophilus*, prevalence, 95% confidence interval (CI) and diagnostic methods were extracted from each article (if available). In the case of described co-infection in lungs, the number and name of co-infecting nematodes were extracted. If some information was not available in the text, it was categorized as “not available”. Data were extracted using Microsoft Excel (Microsoft Office Professional Plus 2019; Microsoft Corp., Redmond, WA, USA). The database containing the extracted information was independently verified by two authors (MSP and JK).

### Quality assessment

The quality assessment score of all included studies was assessed independently by two researchers using the Newcastle‒Ottawa Scale (NOS) according to the Cochrane Handbook for Systematic Reviews [43]. The NOS was modified for use in an animal model.

### Statistical analysis

All calculations necessary for the meta-analysis were conducted in RStudio environment, using the R language version 4.2.1. [44]. Calculations were performed separately for each animal group: dogs (faecal samples), cats (faecal samples, lungs), foxes (faecal samples, lungs) and wild animals (faecal samples, lungs). The metafor package was used to compile results [45]. To determine the heterogeneity of the samples for different subgroups, the Cochran Q-test was applied with significance level of alpha = 0.05. Due to the high heterogeneity of the studies analysed, which involved dogs (faecal samples), cats (faecal samples and lungs) and foxes (faecal samples and lungs), the random-effects model with the restricted maximum likelihood estimator (REML) was applied [46, 47]. For subgroup analysis of small numbers of studies (wild animals—faecal and lung samples), where effect is the same across studies, the fixed-effects model was used [48]. The *I*^2^ value, the percentage of variation in a study that is due to heterogeneity rather than chance, was determined. The average prevalence and 95% CIs were calculated using the binom package [49], which implements a modified Wilson method interval (corrected for Newcombe continuity) [50]. To visualize the results of the analysis, a forest plot was produced in R using the forestplot package [51]. Differences in prevalence were calculated using a Chi-square test (or Chi-square with Yates correction), with a significance level of *P* < 0.05 applied, in Statistica 10 (StatSoft Polska, Kraków, Poland).

## Results

### Literature search summary

The database search identified 606 articles, of which 183 records were removed by EndNote 20 because of duplications, leaving 423 potentially substantial articles for further evaluation. Of these 423 articles, 215 were excluded based on the screening of titles and abstracts; the remaining 208 articles were assessed for eligibility and subjected to full-text inspection. Of these 208 articles, 67 were deemed ineligible studies and excluded from the systematic review due to non-compliance with the pre-established inclusion criteria. Ultimately, a total of 141 studies were included in the qualitative and quantitative (meta-analysis) synthesis. The flow diagram shown in Fig. 1 represents the database search algorithm, with presentation of the study selection process. The included studies are listed in Additional file 1: Table S1, and the excluded studies are listed in Additional file 2: Table S2. The quality assessment of the included studies was accomplished with the modified NOS, resulting in the allocation of rating to each individual study that ranged from four to seven stars.

Studies describing the occurrence of *E. aerophilus* included in the qualitative synthesis and meta-analysis were available from 38 countries all around the world (Table 1). Most studies came from Italy (21 articles) and Spain (13 articles).

**Table 1:** List of countries and articles describing the occurrence of *Eucoleus aerophilus* per country included in the systematic review

Number	Country	Number of articles
1	Albania	1
2	Australia	4
3	Austria	2
4	Belgium	1
5	Bolivia	1
6	Bosnia and Herzegovina	1
7	Bulgaria	2
8	Canada	6
9	Chile	1
10	Croatia	1
11	Denmark	8
12	Estonia	1
13	France	1
14	Germany	5
15	Greece	1
16	Hungary	3
17	Iceland	1
18	India	2
19	Iran	2
20	Italy	21
21	Japan	1
22	Latvia	1
23	Lithuania	2
24	Norway	1
25	Poland	7
26	Portugal	2
27	Republic of Ireland	3
28	Romania	5
29	Russia	1
30	Serbia	6
31	Slovakia	2
32	Spain	13
33	Switzerland	2
34	The Netherlands	1
35	Turkey	1
36	UK	3
37	Uruguay	1
38	USA	7

### Findings from the meta-analysis of prevalence values

Studies included in the analysis presented results obtained by using microscopic methods (flotation) and necropsy, often additionally confirmed by PCR and sequencing (if available), including on the detection of *E. aerophilus* in dog faecal samples (24 articles), in the lungs of cats (7 articles), in cat faecal samples (27 articles), in the lungs of foxes (37 articles), in fox faecal samples (10 articles), in the lungs of wild animals (35 articles) and in wild animal faecal samples (15 articles). Due to the occurrence of *E. aerophilus* in many different species and different matrices (lungs and faeces), we decided to conduct a meta-analysis separately for each species with a given matrix. The above-mentioned studies included in the analysis were published from 1973 to the end of 2022.

#### Occurrence of* E. aerophilus* in fox lungs

**Fig. 2:**
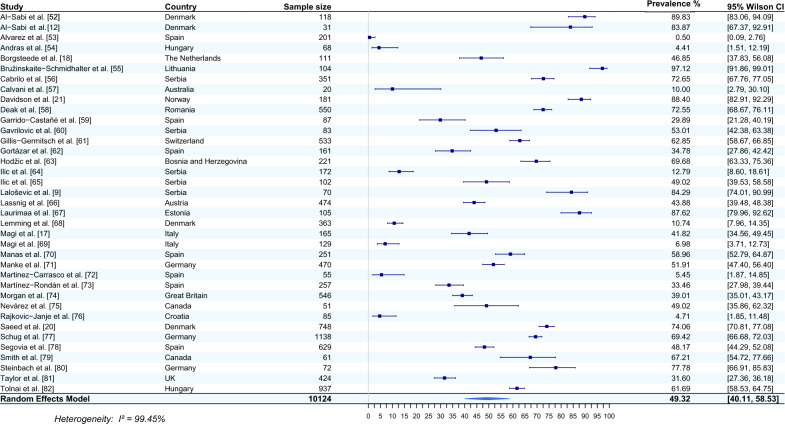
Forest plot of the random effects model of *E. aerophilus* prevalence (%) calculated based on the results from fox lungs. Squares correspond to the prevalence of *E. aerophilus* in individual studies; horizontal lines correspond to 95% Wilson confidence intervals (%) of the prevalence from individual studies; the diamond corresponds to the average prevalence calculated using the random effects model.* I*^*2*^ Statistic that describes the percentage of variation in study that is due to heterogeneity rather than chance

Thirty-seven studies from 19 countries that reported the occurrence of *E. aerophilus* in the lungs of foxes were included in the meta-analysis [9, 12, 17, 18, 20, 21, 52–82]. The average prevalence of this nematode, using a random effects model, was estimated based on a total of 10,124 sampled foxes and was 49.32% (95% CI 40.11–58.53). The heterogeneity was very high, 99.45% (Fig. 2). The highest prevalence of *E. aerophilus* was recorded in samples from Lithuania (97.12%, 95% CI 91.86–99.01) [55], Denmark (89.83%, 95% CI 83.06–94.09) [52] and Norway (88.40%, 95% CI 82.91–92.29) [21]. The lowest prevalence of *E. aerophilus* was recorded in samples from Spain (0.5%, 95% CI 0.09–2.76) [53], Hungary (4.41%, 95% CI 1.51–12.19) [54] and Croatia (4.71%, 95% CI 1.85–11.48) [76].

#### Occurrence of* E. aerophilus *in fox faeces

**Fig. 3:**
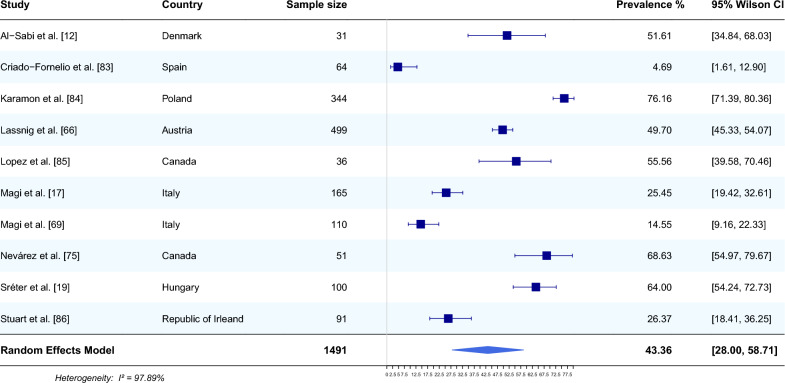
Forest plot of the random effects model of *E. aerophilus* prevalence (%) calculated based on results from fox faeces. Squares correspond to the prevalence of *E. aerophilus* in individual studies; horizontal lines correspond to 95% Wilson confidence intervals (%) of the prevalence from individual studies; the diamond corresponds to the average prevalence calculated using the random effects model.* I*^*2*^ Statistic that describes the percentage of variation in study that is due to heterogeneity rather than chance

Ten studies from eight countries that reported the occurrence of *E. aerophilus* in the faeces of foxes were included in the meta-analysis [12, 17, 19, 66, 69, 75, 83–86]. The average prevalence of this nematode, using a random effects model, was estimated based on a total of 1,491 sampled foxes and was 43.36% (95% CI 28.00–58.71). The heterogeneity was 97.89% (Fig. 3). The highest prevalence of *E. aerophilus* was recorded in Poland (76.16%, 95% CI 71.39–80.36) [84] and Canada (68.63%, 95% CI 54.97–79.67) [75]. The lowest prevalence of *E. aerophilus* was recorded in Spain (4.69%, 95% CI 1.61–12.90) [83].

#### Occurrence of* E. aerophilus* in cat lungs

**Fig. 4:**
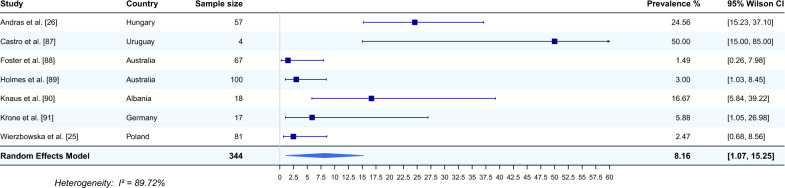
Forest plot of the random effects model of *E. aerophilus* prevalence (%) calculated based on the results from cat lungs. Squares correspond to the prevalence of *E. aerophilus* in individual studies; horizontal lines correspond to 95% Wilson confidence intervals (%) of the prevalence from individual studies; the diamond corresponds to the average prevalence calculated using the random effects model.* I*^*2*^ Statistic that describes the percentage of variation in study that is due to heterogeneity rather than chance

Seven studies from six countries that reported the occurrence of *E. aerophilus* in the lungs of cats were included in the meta-analysis [25, 26, 87–91]. The average prevalence of this nematode, using a random effects model, was estimated based on a total of 283 sampled cats and was 8.16% (95% CI 1.07–15.25). The heterogeneity was 89.72% (Fig. 4). The highest prevalence of *E. aerophilus* was recorded in Uruguay (50%, 95% CI 15.00–85.00) [87], and the lowest prevalence of *E. aerophilus* was recorded in Australia (1.49%, 95% CI 0.26–7.98) [88].

#### Occurrence of* E. aerophilus* in cat faeces

**Fig. 5:**
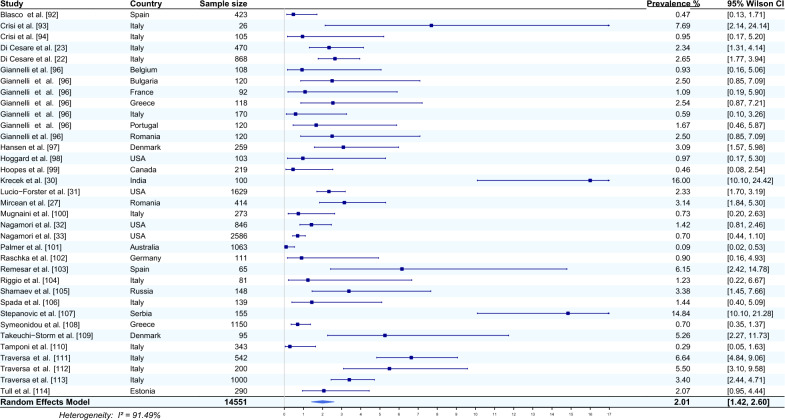
Forest plot of the random effects model of *E. aerophilus* prevalence (%) calculated based on the results from cat faeces. Squares correspond to the prevalence of *E. aerophilus* in individual studies; horizontal lines correspond to 95% Wilson confidence intervals (%) of the prevalence from individual studies; the diamond corresponds to the average prevalence calculated using the random effects model.* I*^*2*^ Statistic that describes the percentage of variation in study that is due to heterogeneity rather than chance

Twenty-nine studies from 16 countries that reported the occurrence of *E. aerophilus* in the faeces of cats were included in the meta-analysis [23, 27, 30–33, 92–114]. The average prevalence of this nematode, using a random effects model, was estimated based on a total of 14,551 sampled cats and was 2.01% (95% CI 1.42–2.60). The heterogeneity was 91.49% (Fig. 5). The highest prevalence of *E. aerophilus* was recorded in India (16%, 95% CI 10.10–24.42) [30], and the lowest prevalence of *E. aerophilus* was recorded in Australia (0.09%, 95% CI 0.02–0.53) [101].

#### Occurrence of* E. aerophilus* in dog faeces

**Fig. 6:**
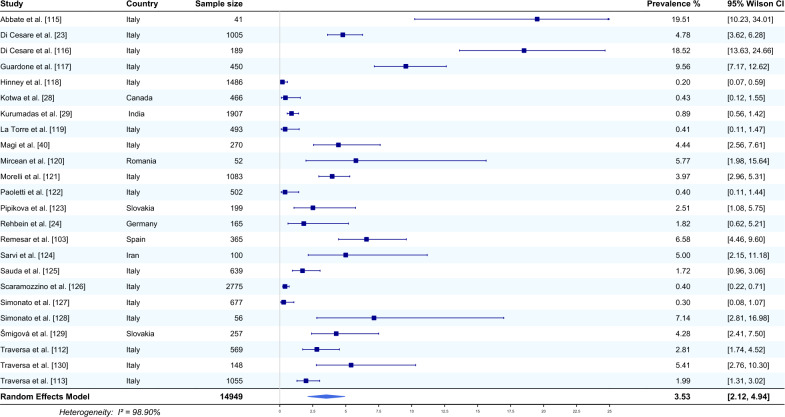
Forest plot of the random effects model of *E. aerophilus* prevalence (%) calculated based on the results from faeces of dogs. Squares correspond to the prevalence of *E. aerophilus* in individual studies; horizontal lines correspond to 95% Wilson confidence intervals (%) of the prevalence from individual studies; the diamond corresponds to the average prevalence calculated using the random effects model.* I*^*2*^ Statistic that describes the percentage of variation in study that is due to heterogeneity rather than chance

Twenty-four studies from eight countries that reported the occurrence of *E. aerophilus* in the faeces of dogs were included in the meta-analysis [23, 24, 28, 29, 40, 103, 112, 113, 115–130]. The average prevalence of this nematode, using a random effects model, was estimated based on a total of 14,949 sampled dogs and was 3.53% (95% CI 2.12–4.94). The heterogeneity was 98.90% (Fig. 6). The highest prevalence of *E. aerophilus* was recorded in Italy (19.51%, 95% CI 10.23–34.01) [115]; 18.52%, 95% CI 13.63–24.66) [116]). The lowest prevalence of *E. aerophilus* was also recorded in Italy (0.2%, 95% CI 0.07–0.59) [118]; 0.3%, 95% CI 0.08–1.07) [127].

#### Occurrence of* E. aerophilus* in wild animal faeces

Fifteen studies from eight countries that reported the occurrence of *E. aerophilus* in the faeces of 10 wild animal species were included in this systematic review [86, 131–144]. Due to the small number of studies included in the analysis, the average prevalence of *E. aerophilus* was estimated using a fixed-effects model. Wolf (*Canis lupus*) and badger (*Meles meles*) were the most frequently described species, with five and three articles, respectively. The highest prevalence of *E. aerophilus* was recorded in the crab-eating fox (*Cerdocyon thous*) from Bolivia [135], at 33.33%. The lowest prevalence was observed in single studies on the brown bear (*Ursus arctos marsicanus*) (2.50%) [134] and the lynx (*Lynx lynx*) (5%) [140]. The results are presented in Table 2.

**Table 2:** Average prevalence of *E. aerophilus* in faeces of wild animals calculated using the fixed-effects model

Species	No. of studies included	Average prevalence (%)	95% CI	Country	References
Arctic fox (*Vulpes/Alopex lagopus*)	1	6.00^a^	1.56–17.54	Iceland	Skírnisson et al. [131]
Badger (*Meles meles*)	3	8.02	5.86–10.17	Republic of Ireland	Byrne et al. [132]
					Kelly et al. [133]
					Stuart et al. [86]
Brown bear (*Ursus arctos marsicanus*)	1	2.50^a^	0.43–9.57	Italy	Paoletti et al. [134]
Crab-eating fox (*Cerdocyon thous*)	1	33.33^a^	1.76–87.47	Bolivia	Fiorello et al. [135]
European wildcat (*Felis silvestris*)	2	26.17	19.81–32.54	Greece	Diakou et al. [136]
				Italy	Napoli et al. [137]
Geffroy's cat (*Oncifelis geoffroyi*)	1	12.50^a^	0.66–53.32	Bolivia	Fiorello et al. [135]
Hedgehog (*Erinaceus* sp.)	2	17.94	13.52–22.36	Germany	Barutzki et al. [138]
				Poland	Mizgajska-Wiktor et al. [139]
Lynx (*Lynx lynx*)	1	5.00^a^	1.86–11.83	Poland	Szczęsna et al. [140]
Ocelot (*Leopardus pardalis*)	1	20.00^a^	3.54–55.78	Bolivia	Fiorello et al. [135]
Wolf (*Canis lupus*)	5	16.19	3.95–28.43	Italy	Di Francesco et al. [141]
					Paoletti et al. [134]
				Portugal	Figueiredo et al. [142]
				Poland	Popiołek et al. [143]
					Szafrańska et al. [144]

*CI* Confidence interval

^a^Values is prevalence estimated from only one study, not average prevalence

#### Occurrence of* E. aerophilus* in wild animal lungs

Thirty-five studies from 20 countries that reported the occurrence of *E. aerophilus* in the lungs of 18 species of wild animals were included in this systematic review [55, 67, 68, 91, 136, 145–170]. Due to the small number of studies included in the analysis, the average prevalence of *E. aerophilus* was estimated using a fixed-effects model. The European wildcat (*Felis silvestris*) and raccoon dog (*Nyctereutes procyonoides*) were the most frequently described species, with six and five articles, respectively. The highest prevalence of *E. aerophilus* was recorded in the badger (*Meles meles*) and European pine marten (*Martes martes*), at 66.67% and 50.98%, respectively. The lowest prevalence was observed in the American marten (*Martes americana*) (0.99%) and raccoon dog (*Nyctereutes procyonoides*) (3.03%). The results are presented in Table 3.

**Table 3:** Average prevalence of *E. aerophilus* in the lungs of wild animals was calculated using the fixed-effects model

Species	No. of studies included	Average prevalence (%)	95% CI	Country	References
American marten (*Martes americana*)	1	0.99^a^	0.32–2.69	Canada	Seville et al. [145]
American mink (*Neovision vision*)	1	13.56^a^	6.45–25.53	Lithuania	Nugaraitė et al. [146]
Badger (*Meles meles*)	1	66.67^a^	24.11–94.00	Hungary	Takacs et al. [147]
Beech marten (*Martes foina*)	3	11.13	3.79–18.46	Denmark	Lemming et al. [68]
				Bulgaria	Panayotova-Pancheva et al. [148]
				Japan	Sato et al. [149]
Bobcat (*Lynx rufus*)	1	4.00^a^	1.04–12.03	USA	Tiekotter et al. [150]
Coyote (*Canis latrans*)	2	12.65	10.23–15.08	USA	Morrison et al. [151]
					Morrison et al. [152]
European otter (*Lutra lutra*)	1	10.00^a^	4.96–18.59	Denmark	Takeuchi-Storm et al. [153]
European pine marten (*Martes martes*)	1	50.98^a^	40.95–60.94	Spain	Segovia et al. [154]
European polecat (*Mustela putorius*)	1	34.62^a^	17.95–55.64	Lithuania	Nugaraitė et al. [146]
European wildcat (*Felis silvestris*)	6	26.98	18.92–35.03	Romania	Deak et al. [155]
				Greece	Diakou et al. [136]
				Italy	Falsone et al. [156]
					Veronesi et al. [158]
				Germany	Krone et al. [91]
				Hungary	Takacs et al. [157]
Guigna (*Leopardus guigna*)	1	9.38^a^	2.46–26.17	Chile	Acuña-Olea et al. [159]
Hedgehog (*Erinaceus* sp.)	4	5.27	3.18–7.35	Turkey	Cirak et al. [160]
				UK	Gaglio et al. [161]
				Iran	Naem et al. [162]
				Denmark	Rasmussen et al. [163]
Iberian lynx (*Lynx pardinus*)	1	12.50^a^	0.66–53.32	Spain	Torres et al. [164]
Iberian wolf (*Canis lupus signatus*)	2	4.39	0.88–7.89	Spain	Estevez-Sanchez et al. [168]
					Martínez-Rondán et al. [73]
Jackal (*Canis aureus*)	2	14.57	6.58–22.29	Serbia	Čabrilo et al. [56]
				Hungary	Takacs et al. [169]
Mustelidae	1	14.52^a^	9.06–22.24	France	Torres et al. [170]
Raccoon dog (*Nyctereutes procyonoides*)	5	3.03	1.96–4.11	Lithuania	Bružinskaitė-Schmidhalter et al. [55]
				Estonia	Laurimaa et al. [67]
				Denmark	Lemming et al. [68]
				USA	Richardson et al. [165]
				Germany	Thiess et al. [166]
Wolf (*Canis lupus)*	1	36.36^a^	20.96–54.85	Latvia	Bagrade et al. [167]

*CI* Confidence interval

^a^Values is prevalence estimated from only one study, not average prevalence

#### Impact of different factors on the occurrence of* E. aerophilus* in animals

During the data extraction, several factors emerged that could potentially affect the occurrence of the described lungworm in the analysed subgroups of animals, including age, sex and/or environmental/living conditions. All of these factors were divided into specific species and matrices, and statistically significant differences in prevalence were noted (if available).

##### Impact of age on the occurrence of *E. aerophilus* in fox lungs

Data describing the impact of age on the occurrence of *E. aerophilus* in the lungs of foxes were available from seven studies [20, 58, 59, 64, 70, 72, 73]. Statistically significant differences in prevalence (*P* < 0.05) occurred in only two cases: in Serbia, in the study by Ilić et al. [64], and in Spain, in the study by Manas et al. [70]. The results are presented in Table 4.

**Table 4:** Impact of age on the occurrence of *E. aerophilus* in fox lungs

Country	Sample type	Young foxes (< 12 months)			Adult foxes (> 12 months)			References
		*n*^a^	Prevalence (%)	95% CI	*n*^a^	Prevalence (%)	95% CI	
Romania	Lungs	180	74.44	67.42–80.64	370	71.62	66.82–75.97	Deak et al. [58]
Spain	Lungs	21	19.05	5.4–41.9	66	33.33	22.2–46	Garrido-Castañé et al. [59]
Serbia	Lungs	69	0.00*	0.00	103	21.36*	14.55–30.23	Ilić et al. [64]
Spain	Lungs	58	17.24*	9.64–28.91	193	71.50*	64.76–77.4	Manas et al. [70]
Spain	Lungs	26	0.00	0.00	29	10.34	3.58–26.38	Martinez-Carrasco et al. [72]
Spain	Lungs	71	25.35	16.68–36.55	186	36.56	29.98–43.69	Martínez-Rondán et al. [73]
Denmark	Lungs	468	75.43	71.33–79.11	280	71.79	66.25–76.74	Saeed et al. [20]

*CI* Confidence interval

*Statistically significant differences in prevalence at *P* < 0.05

^a^Size of the study population

##### Impact of sex on the occurrence of *E. aerophilus* in fox lungs

Data describing the impact of sex on the occurrence of *E. aerophilus* in the lungs of foxes were available from seven studies [20, 21, 58, 59, 63, 73, 74]. Statistically significant differences in prevalence (*P* < 0.05) occurred in only two cases: in Romania, in the study by Deak et al. [58], and the UK, in the study by Morgan et al. [74]. The results are presented in Table 5.

**Table 5:** Impact of sex on the occurrence of *E. aerophilus* in fox lungs

Country	Sample type	Male foxes			Female foxes			References
		*n*^a^	Prevalence (%)	95% CI	*n*^a^	Prevalence (%)	95% CI	
Norway	Lungs	111	89.19	82.05–93.71	70	87.14	77.33–93.08	Davidson et al. [21]
Romania	Lungs	315	77.46*	72.53–81.73	235	65.96*	59.51–71.99	Deak et al. [58]
Spain	Lungs	46	32.61	19.5–48.0	41	26.83	14.2–42.9	Garrido-Castañé et al. [59]
Bosnia and Herzegovina	Lungs	123	73.17	64.73–80.21	98	65.31	55.47–73.99	Hodžić et al. [63]
Spain	Lungs	149	34.23	27.09–42.16	108	32.41	24.32–41.71	Martínez-Rondán et al. [73]
UK	Lungs	143	60.84*	52.66–68.46	128	40.63*	32.52–49.29	Morgan et al. [74]
Denmark	Lungs	381	74.54	69.94–78.65	367	73.57	68.83–77.28	Saeed et al. [20]

*CI* Confidence interval

*Statistically significant differences in prevalence at *P* < 0.05

^a^Size of the study population

##### Impact of age on the occurrence of *E. aerophilus* in cat faeces

Data describing the impact of age on the occurrence of *E. aerophilus* in the faecal samples of cats were available from seven studies. Statistically significant differences in prevalence (*P* < 0.05) occurred in only one case: in the USA, in the study by Nagamori et al. [33]. The results are presented in Table 6.

**Table 6:** Impact of age on the occurrence of *E. aerophilus* in cat faeces

Country	Sample type	Young cats (< 12 months)			Adult cats (> 12 months)			References
		*n*^a^	Prevalence (%)	95% CI	*n*^a^	Prevalence (%)	95% CI	
Italy	Faecal samples	281	2.85	1.45–5.52	587	2.56	1.56–4.18	Di Cesare et al. [22]
USA	Faecal samples	66	0.00	0	37	2.70	0.48–13.82	Hoggard et al. [98]
Romania	Faecal samples	169	2.37	0.93–5.93	245	3.67	1.94–6.83	Mircean et al. [27]
USA	Faecal samples	458	1.53	0.74–3.12	388	1.29	0.4–3	Nagamori et al. [32]
USA	Faecal samples	1245	9.00*	7.53–40.72	504	5.95*	0.2–1.7	Nagamori et al. [33]
Italy	Faecal samples	211	0.00	0	132	0.76	0.13–4.17	Tamponi et al. [110]
Estonia	Faecal samples	120	1.67	0.46–5.88	170	2.35	0.92–5.89	Tull et al. [114]

*CI* Confidence interval

*Statistically significant differences in prevalence at *P* < 0.05

^a^Size of the study population

##### Impact of sex on the occurrence of *E. aerophilus* in cat lungs and faeces

Data describing the impact of sex on the occurrence of *E. aerophilus* in the lungs and faecal samples of cats were available from five studies. There were no statistically significant differences in prevalence (*P* > 0.05). The results are presented in Table 7.

**Table 7:** Impact of sex on the occurrence of *E. aerophilus* in cat lungs and faeces

Country	Sample type	Male cats			Female cats			References
		*n*^a^	Prevalence (%)	95% CI	*n*^a^	Prevalence (%)	95% CI	
Germany	Lungs	10	10.00	1.79–40.41	7	0.00	0	Krone et al. [91]
Poland	Lungs	53	1.89	0.33–9.95	28	3.57	0.63–17.71	Wierzbowska et al. [25]
Italy	Faecal samples	436	2.52	1.79–40.41	429	2.80	1.61–4.83	Di Cesare et al. [22]
Romania	Faecal samples	187	2.67	0.33–9.95	277	2.89	1.47–5.6	Mircean et al. [27]
Estonia	Faecal samples	104	0.00	0	148	2.70	1.05–6.74	Tull et al. [114]

*CI* Confidence interval

^a^Size of the study population

##### Impact of environmental conditions on the occurrence of *E. aerophilus* cat lungs and faeces

Data describing the impact of the environmental conditions on the occurrence of *E. aerophilus* in the lungs and faecal samples of cats were available from three studies. Statistically significant differences in prevalence (*P* < 0.05) occurred in only one case: in Romania, in the study by Mircean et al. [27]. The results are presented in Table 8.

**Table 8:** Impact of environmental conditions on the occurrence of *E. aerophilus* in cat lungs and faeces

Country	Sample type	Urban cats			Rural cats			References
		*n*^a^	Prevalence (%)	95% CI	*n*^a^	Prevalence (%)	95% CI	
Poland	Lungs	33	3.0	0.5–15.3	48	2.1	0.4–10.9	Wierzbowska et al. [25]
Romania	Faecal samples	285	0.4*	0.1–2.0	128	9.4*	5.5–15.7	Mircean et al. [27]
Estonia	Faecal samples	130	0.8	0.1–4.2	160	3.1	1.4–7.1	Tull et al. [114]

*CI* Confidence interval

*Statistically significant differences in prevalence at *P* < 0.05

^a^Size of the study population

##### Impact of age on the occurrence of *E. aerophilus* in dog faeces

Data describing the impact of age on the occurrence of *E. aerophilus* in faecal samples of dogs were available from four studies. Statistically significant differences in prevalence (*P* < 0.05) occurred in only one case: in Italy, in the study by Guardone et al. [117]. The results are presented in Table 9.

**Table 9:** Impact of age on the occurrence of *E. aerophilus* in dog faeces

Country	Sample type	Young dogs (< 12 m)			Adult dogs (> 12 m)			References
		*n*^a^	Prevalence (%)	95% CI	*n*^a^	Prevalence (%)	95% CI	
Italy	Faecal samples	57	21.05*	12.47–33.28	393	7.89*	5.61–10.98	Guardone et al. [117]
India	Faecal samples	1071	1.12	0.64–1.95	836	0.60	0.26–0.14	Kurumadas et al. [29]
Iran	Faecal samples	21	4.76	0.85–22.67	79	5.06	1.98–12.3	Sarvi et al. [124]
Slovakia	Faecal samples	89	7.87	3.87–15.36	168	2.38	0.93–5.96	Šmigová et al. [129]

*CI* Confidence interval

*Statistically significant differences in prevalence at *P* < 0.05

^a^Size of the study population

##### Impact of sex on the occurrence of *E. aerophilus* in dog faeces

Data describing the impact of sex on the occurrence of *E. aerophilus* in faecal samples of dogs were available from three studies. Statistically significant differences in prevalence (*P* < 0.05) occurred in only one case: in Italy, in the study by Guardone et al. [117]. The results are presented in Table 10.

**Table 10:** Impact of sex on the occurrence of *E. aerophilus* in dog faeces

Country	Sample type	Male dogs			Female dogs			References
		*n*^a^	Prevalence (%)	95% CI	*n*^a^	Prevalence (%)	95% CI	
Italy	Faecal samples	260	6.92*	4.42–10.67	190	13.16*	9.08–18.7	Guardone et al. [117]
India	Faecal samples	915	0.87	0.44–1.71	992	0.91	0.48–1.72	Kurumadas et al. [29]
Iran	Faecal samples	56	7.14	2.81–16.97	44	2.27	0.4–1.18	Sarvi et al. [124]

*CI* Confidence interval

*Statistically significant differences in prevalence at *P* < 0.05

^a^Size of the study population

### Co-infections with other lungworms in a group of *E. aerophilus*-positive foxes

Data describing co-infections with other lungworms in a group of *E. aerophilus*-positive foxes were available from 11 studies [12, 17, 21, 52, 58–61, 63, 77, 81]. Simultaneously with *E. aerophilus*, species such as *Crenosoma vulpis*, *Angiostrongylus vasorum*, *Eucoleus boehmi* and *Filaroides* spp. were detected in the lungs of foxes. The most frequently detected co-infection was with *C. vulpis*, with a frequency ranging from 5.1% to 53.8%. The less frequently detected co-infection was with *E. boehmi*, with a frequency ranging from 14.9% to 18.8%. Triple co-infections (*E. aerophilus* + *C. vulpis* + *A. vasorum*) were described in eight studies, and a quadruple co-infection was detected in only one study (*E. aerophilus* + *C. vulpis* + *A. vasorum* + *Filaroides* spp.). The results are presented in Table 11.

**Table 11:** Co-infections in a group of *E. aerophilus*-positive foxes with other lungworms

References	*n*^a^	No. of* E. aerophilus*-positive foxes	% of co-infections in a group of *E. aerophilus*-positive foxes				
			EA + CV	EA + AV	EA + EB	EA + CV + AV	EA + CV + EB
Al-Sabi et al. [52]	118	106	13.2	34.9	n.a	2.8	n.a
Al-Sabi et al. [12]	31	26	23.1	23.8	n.a	11.5	n.a
Davidson et al. [21]	181	160	22.5	n.a	18.8	n.a	33.8
Deak et al. [58]	550	399	39.1	n.a	n.a	n.a	n.a
Garrido-Castañé et al. [59]	87	26	53.8	0	n.a	3.8	n.a
Gavrilović et al. [60]	83	44	34.1	4.5	n.a	4.5	n.a
Gillis-Germitsch et al. [61]	533	335	5.1	47.8	n.a	7.2	n.a
Hodžić et al. [63]	221	154	39.6	n.a	14.9	n.a	10.4
Magi et al. [17]	165	69	0	55.1	n.a	20.3^b^	n.a
Schug et al. [77]	1138	790	34.2	9.4	n.a	5.6	n.a
Taylor et al. [81]	424	134	8.2	12.7	n.a	4.5	n.a

*AV* *Angiostrongylus vasorum*,* CV* *Crenosoma vulpis*,* EA E. aerophilus*,* EB Eucoleus boehmi*,* n.a.* not available

^a^Size of the study population

^b^Additionally, quadruple co-infection was detected with *Filaroides* spp. with a prevalence in a group of foxes positive for *E. aerophilus* of 2.9%

## Discussion

*Eucoleus aerophilus* is a zoonotic parasite affecting both domestic and wild animals, as well as humans. It causes respiratory capillariosis, with a subclinical course in most cases, but it can occasionally lead to respiratory distress ranging from mild disease to severe and potentially fatal pneumonia [13]. Despite steadily increasing knowledge of the morphology of this neglected parasite, many aspects are still largely unknown. Systematic epidemiological reviews or meta-analyses on the occurrence of *E. aerophilus* in wild and domestic animals have not yet been carried out. To the best of our knowledge, we present here the first systematic review to evaluate and summarize existing knowledge on the occurrence and prevalence of *E. aerophilus* in wild and domestic animals originating from different geographical locations worldwide. In our work, we used a comprehensive approach to extract eligible articles on *E. aerophilus* detection. Data from almost 50 years of research in this field, from 38 countries and describing 36 animal species, are summarized in this review. The information thus obtained was used to compile tables on the prevalence of this nematode, focusing on identifying data gaps and promoting future research directions in this area.

The most common host and reservoir of *E. aerophilus* is the red fox. The prevalence of this parasite in red foxes is usually high, with a wide geographic distribution (Figs. 2, 3). It is therefore not surprising that the largest number of articles on *E. aerophilus* detection concerned this group of animals, with 37 and 10 articles reporting the occurrence of *E. aerophilus* in the lungs of foxes (Fig. 2) and in the faeces (Fig. 3), respectively. The included studies refer to almost all European countries but also to Canada [75] and/or Australia [57]. The results obtained in this meta-analysis revealed that the average prevalence of *E. aerophilus* detected in fox lungs by necropsy was 49.32% (95% CI 40.11–58.53), with the highest prevalence of 97.12% (95% CI 91.86–99.01) reported in Lithuania [55] and the lowest prevalence of 0.50% (95% CI 0.09–2.76) reported in Spain [53]. Comparing the summary results for foxes from postmortem lung examination with faecal examination, we noted a rather similar percentage of positive results, with the average prevalence of *E. aerophilus* based on flotation being 43.36% (95% CI 28.00–58.71). The analysis of the results obtained by individual studies indicated that the results are quite diverse. For example, in the study by Al-Sabi et al. [12], the recovery of lungworm eggs with faecal examination was 32% lower than the postmortem recovery of *E. aerophilus* worms from lungs [12]. Notwithstanding, Nevárez et al. [75], in their study on the distribution of *E. aerophilus* in lungs, reported a 49% prevalence, while in faecal examination, 68.6% of foxes were positive for *E. aerophilus*. Such discrepancies can be explained by damage to eggs during the freezing and thawing cycles before testing due to the use of inappropriate flotation medium or techniques [171] or by the intermittent and irregular patterns of egg excretion [2]. Another factor that may have contributed to the disparity in the results is the fact that individual species of parasites have diversified distributions in the lungs. According to the study by Nevárez et al. [75], *E. aerophilus* is mainly restricted to the large bronchi of caudal lobes. Moreover, faecal examination can lead to misleading results, especially in relation to the whipworm *T. vulpis*, which has similar morphological and morphometric features to *E. aerophilus* [11].

An increase in the red fox population, coupled with a decrease in natural habitats due to progressive urbanization, plays a key role in the spread and transmission of *E. aerophilus* to domestic animals [5, 13]. In contrast to foxes, where the study of lungworms is mostly carried out with the use of necropsy, in domestic animals (such as dogs or cats), such infections are investigated principally by examining faecal samples, with the flotation test or by PCR. In this systematic review, a meta-analysis of articles referring to cats revealed that the detection of *E. aerophilus* from lungs gave a much higher average prevalence (8.16%) than detection from faecal samples (2.01%). It is worth mentioning that the vast majority of articles on the detection of *E. aerophilus* in cats tested faecal samples (29 studies) (Fig. 5), and only seven studies reported the occurrence of *E. aerophilus* in the lungs (Fig. 4). The highest prevalence of *E. aerophilus* was recorded in Uruguay, at 50% [87] (detection in lungs), and in India, at 16% [30] (detection in faeces), and the lowest prevalence of *E. aerophilus* was recorded in Australia, at 1.49% (detection in lungs) [88] and 0.09% (detection in faeces) [101].

In our review, studies in dogs referred only to faecal samples (24 studies) (Fig. 6), as no article describing the detection of *E. aerophilus* in the lungs of these companion animals was available. The vast majority of articles (16 studies) originated from Italy [23, 40, 112, 113, 115–119, 121, 122, 125–128, 130], but there were also studies from other European countries [24, 120, 123, 129] or other parts of the globe, such as Canada [28], India [29] and Iran [124]. Meta-analysis of the data reported on this species revealed that the average prevalence of *E. aerophilus* was 3.53% (95% CI 2.12–4.94), which was similar to that found in cats. The highest and lowest prevalence of *E. aerophilus* in dogs was recorded in Italy, at 19.51% [115] and 0.09% [118], respectively. Despite increased concern for companion animal health and the use of highly efficient antiparasitic drugs, recent studies conducted throughout the world have shown that infections caused by lungworms remain a common occurrence in both dogs and cats. Nevertheless, the reported prevalence of this parasite is much lower in dogs and cats than in foxes. This difference may be related to an underestimation of lung capillariosis by veterinarians due to the lack of basic parasitological research conducted in this direction and, as already mentioned, to misdiagnosis of *T. vulpis* infection upon microscopic examination [11].

In addition to foxes, *E. aerophilus* has been reported in many different wild species, shown in Tables 2 and 3. The most frequently reported wild species infected with *E. aerophilus* in the lungs were the European wildcat [91, 136, 155–158], raccoon dog [55, 68, 165, 166, 172] and hedgehog [160–163]. On faecal examination, the most frequently reported species testing positive for *E. aerophilus* were the wolf [134, 141–144] and badger [86, 132, 133]. Analysis of the results from lungs of wild animals revealed an overall prevalence of *E. aerophilus* ranging from 66.67% [147] to 0.99% [145]; from faecal samples, overall prevalence ranged from 33.33% [135] to 2.50% [134]. These epidemiological data strongly support the hypothesis that wild carnivores act as the main definitive hosts for the analysed nematode [173] and are consistent with the results from foxes. The sharing of habitat between domestic and wild animals facilitates the transmission of parasites between them. The large number of studies on the occurrence of *E. aerophilus* in the lungs of wild animals compared to domestic animals is because it was possible to collect the carcasses of animals killed in road accidents or by hunters, as well as other reasons.

It should be emphasized that all of the prevalence analyses in this review were performed globally for animal species. Analysed studies were divided into subgroups that considered dogs (faecal samples), cats (faecal samples and lungs), foxes (faecal samples and lungs) and wild animals (faecal samples and lungs) separately. Taking into account the large variation in the applied flotation variants that emerged during data extraction, we decided to group all flotation results (regardless of the variant) into one group within the animal species in order to be able to perform the analysis. Moreover, differences between regions, countries and groups of animals of the same species were not considered in the analysis of the prevalence. On the other hand, the impact of various factors on the occurrence of *E. aerophilus* in animals was analysed. During the data extraction, several factors emerged that could potentially affect the occurrence of the described lungworm, including age, sex or environmental/living conditions. All of these were categorized into specific species and matrices, and statistically significant differences in prevalence (if available) have been noted.

The data on the impact of host-related factors (age, sex or environmental/living conditions) on the prevalence of *E. aerophilus* vary widely. Analysis of the influence of age on the occurrence of *E. aerophilus* in the lungs of foxes was available from seven studies [20, 58, 59, 64, 70, 72, 73], and only in two cases [64, 70] was it shown that adult foxes were significantly more susceptible to pulmonary capillariosis (Table 4). Nevertheless, the number of examined juveniles was lower than the number of examined adults in almost every case, which could have had a significant impact on the results. Similarly, in the case of the influence of sex on susceptibility to *E. aerophilus* infection, out of the seven analysed articles [20, 21, 58, 59, 63, 73, 74], only two [58, 74] showed statistically significant differences and indicated that males were more susceptible to infection with *E. aerophilus* (Table 5). It is worth mentioning that the number of tested males was definitely higher than that of females. In the case of cats, three factors were analysed, namely age, sex and environmental conditions, all of which could influence the occurrence of *E. aerophilus* in lungs and faecal samples. Analysis of seven studies [22, 27, 32, 33, 98, 110, 114] referring to age revealed that only one study [33] reported that young cats were significantly more susceptible to *E. aerophilus* than adult cats (Table 6). No statistically significant differences were found when analysing the impact of sex on the occurrence of *E. aerophilus* in the lungs and faecal samples of cats (Table 7). Analysis of environmental conditions (Table 8) revealed that rural cats are more susceptible to *E. aerophilus* than urban cats [27]. This finding is associated with the outdoor access of cats in rural areas to wilderness areas. In the case of dogs, host-related factors, such as age and sex, were analysed in relation to the prevalence of *E. aerophilus.* Analysis of the extracted results referring to age (Table 9) indicates that only in the study by Guardone et al. [117] were younger dogs more liable to infection with *E. aerophilus* than adult dogs, which is consistent with the results in cats [33]. Also, female dogs were more vulnerable to lung capillariosis than male dogs [117] (Table 10).

Mixed infections are common among wild animals, which are regarded as potential reservoirs of parasites. In this systematic review, we analysed eleven articles describing co-infections in the lungs of *E. aerophilus*-positive foxes (Table 11) [12, 17, 21, 52, 58–61, 63, 77, 81]. The most frequent infection reported in all analysed studies was *E. aerophilus* + *C. vulpis* with a prevalence in a group of *E. aerophilus*-positive foxes ranging from 5.1% to 53.8%. In contrast to *E. aerophilus*, which is naturally restricted to the large bronchi and the caudal lobes, *C. vulpis* was reported in the small bronchi and bronchioles of all pulmonary lobes [75]. The next most frequent co-infection was with *E. aerophilus* + *A. vasorum*, ranging in a group of *E. aerophilus*-positive foxes from 9.4% to 55.1%, thus occurring at almost the same level as *E. aerophilus* + *C. vulpis*. *Angiostrongylus vasorum*, called the “French heartworm”, parasitizes the right ventricle and pulmonary arteries of canids and is distributed worldwide [74]. In addition to lung examination, the basic diagnosis of *A. vasorum* consists of the detection of larvae in the faeces by the Baermann method or in expectorated mucus, and the faecal flotation method is also used. These methods are laborious and limited due to the periodic excretion of larvae in faeces or bronchial secretions, the small number of larvae and the difficulty in distinguishing *A. vasorum* larvae from larval stages of other lungworms such as *C. vulpis* and *Filaroides* spp. [74]. Triple co-infections with *E. aerophilus* + *C. vulpis* + *A. vasorum* were reported in eight articles [12, 17, 52, 59–61, 77, 81]; additionally, in one article, a quadruple co-infection with *E. aerophilus* + *C. vulpis* + *A. vasorum* + *Filaroides* spp. was detected [17]. Mixed infection with *E. aerophilus* + *E. boehmi* was less frequently reported, and it was possible to extract data from only two articles [21, 63]. *Eucoleus boehmi* occurs in the nasal cavity and sinuses of wild and domestic canids, but its life-cycle is still undetermined. Moreover, in contrast to the investigated parasite, *E. boehmi* does not have zoonotic potential [10].

## Conclusions

*Ecoleus aerophilus* is a nematode with zoonotic potential and worldwide geographical distribution. It affects both wild and domestic animals, causing lung capillariosis. This systematic review confirmed the status of the red fox as the main reservoir and transmitter of *E. aerophilus* and evidenced a higher prevalence of *E. aerophilus* in wild animals than in domestic animals. Wildlife migration and colonization of rural areas increase the transmission of this lungworm between wild and domestic carnivores, but also to humans. Many host-related factors (age, sex, environmental/living conditions) have been investigated in relation to the prevalence of *E. aerophilus*, but they show wide variations, and there is no simple relationship. Furthermore, mixed infections with other pulmonary nematodes, such as *C. vulpis* and/or *A. vasorum*, are reported very frequently, which greatly complicates diagnosis. To summarize, this systematic review focused on identifying data gaps and promoting future research directions in this area.

## Supplementary Information

**Additional file 1: Table S1.** List of included studies.

**Additional file 2: Table S2.** List of excluded studies.

## Data Availability

All data and material are presented in the manuscript and supplementary material. The datasets used and/or analysed during the present study are available from the corresponding author upon reasonable request.

## Abbreviations

CI: Confidence interval
MOOSE: Meta-analysis of observational studies in epidemiology
NOS: Newcastle‒Ottawa Scale
PRISMA: Preferred reporting items for systematic reviews and meta-analysis
REML: Restricted maximum likelihood estimator
