# Supplementary material for: Occurrence of Eucoleus aerophilus in wild and domestic animals: a systematic review and meta-analysis
Source: Parasit Vectors. 2023 Jul 20;16:245. doi: 10.1186/s13071-023-05830-0 (PMC10360280; doi:10.1186/s13071-023-05830-0)
Supplement: Supplementary file 1 — Additional file 1: Table S1. List of included studies. [file 13071_2023_5830_MOESM1_ESM.docx]

**Table S1.** List of included studies.

| NO. | REFERENCES |
| --- | --- |
| 1. | Abbate, J.M.; Napoli, E.; Arfuso, F.; Gaglio, G.; Giannetto, S.; Halos, L.; Beugnet, F.; Brianti, E. Six-month field efficacy and safety of the combined treatment of dogs with Frontline Tri-Act® and NexGard Spectra®. *Parasit Vectors* **2018**, *11*, 425, doi:10.1186/s13071-018-2957-7. |
| 2. | Acuña-Olea, F.; Sacristán, I.; Aguilar, E.; García, S.; López, M.J.; Oyarzún-Ruiz, P.; Brito, J.L.; Fredes, F.; Napolitano, C. Gastrointestinal and cardiorespiratory endoparasites in the wild felid guigna (Leopardus guigna) in Chile: Richness increases with latitude and first records for the host species. *International Journal for Parasitology: Parasites and Wildlife* **2020**, *13*, 13-21, doi:<https://doi.org/10.1016/j.ijppaw.2020.07.013>. |
| 3. | Al-Sabi, M.N.; Halasa, T.; Kapel, C.M. Infections with cardiopulmonary and intestinal helminths and sarcoptic mange in red foxes from two different localities in Denmark. *Acta Parasitol* **2014**, *59*, 98-107, doi:10.2478/s11686-014-0214-6. |
| 4. | Al-Sabi, M.N.S.; Kapel, C.M.O. First report of Eucoleus boehmi in red foxes (Vulpis vulpis) in Denmark, based on coprological examination. *Acta Parasitologica* **2013**, *58*, 570-576, doi:10.2478/s11686-013-0182-2. |
| 5. | Alvarez, F.; Iglesias, R.; Bos, J.; Rey, J.; Sanmartin Durán, M.L. Lung and hearth nematodes in some Spanish mammals. *Wiad Parazytol* **1991**, *37*, 481-490. |
| 6. | Andras, T. Data on the parasitological status of the red fox in Hungary. *Magyar Allatorvosok Lapja* **2001**, *123*, 100-107. |
| 7. | Andras, T.; Peter, T. Data on worm infestation of domestic cats (Felis catus) in Hungarian hunting areas. *Magyar Allatorvosok Lapja* **2002**, *124*, 26-30. |
| 8. | Bagrade, G.; Kirjusina, M.; Vismanis, K.; Ozoliņs, J. Helminth parasites of the wolf Canis lupus from Latvia. *J Helminthol* **2009**, *83*, 63-68, doi:10.1017/s0022149x08123860. |
| 9. | Barutzki, D.; Laubmeier, E.; Forstner, M.J. [Endoparasitic infestation of wild hedgehogs and hedgehogs in human care with a contribution to therapy]. *Tierarztl Prax* **1987**, *15*, 325-331. |
| 10. | Blasco, X.; Salas, A.; Manuelian, C.L.; Torre, C.; Ortuno, A. Intestinal Parasitic Infection in Multi-Cat Shelters in Catalonia. *Israel Journal of Veterinary Medicine* **2017**, *72*, 16-21. |
| 11. | Borgsteede, F.H. Helminth parasites of wild foxes (Vulpes vulpes L.) in The Netherlands. *Z Parasitenkd* **1984**, *70*, 281-285, doi:10.1007/bf00927813. |
| 12. | Bružinskaitė-Schmidhalter, R.; Šarkūnas, M.; Malakauskas, A.; Mathis, A.; Torgerson, P.R.; Deplazes, P. Helminths of red foxes (Vulpes vulpes) and raccoon dogs (Nyctereutes procyonoides) in Lithuania. *Parasitology* **2012**, *139*, 120-127, doi:10.1017/s0031182011001715. |
| 13. | Byrne, R.L.; Fogarty, U.; Mooney, A.; Harris, E.; Good, M.; Marples, N.M.; Holland, C.V. The helminth parasite community of European badgers (Meles meles) in Ireland. *J Helminthol* **2019**, *94*, e37, doi:10.1017/s0022149x19000051. |
| 14. | Čabrilo, O.B.; Simin, V.; Miljević, M.; Čabrilo, B.; Mijatović, D.; Lalošević, D. Respiratory and Cardiopulmonary Nematode Species of Foxes and Jackals in Serbia. *Helminthologia* **2018**, *55*, 213-221, doi:10.2478/helm-2018-0019. |
| 15. | Calvani, N.E.D.; Wright, M.; White, J.; Stepkovitch, B.; Francis, E.; Rivory, P.; Wong, B.; Wilson, T.; Walker, M.; Martin, P.; et al. What the fox? Cryptic Eucoleus [Capillaria] sp. in the respiratory tract of a cat from Australia. *Current Research in Parasitology & Vector-Borne Diseases* **2021**, *1*, 100028, doi:<https://doi.org/10.1016/j.crpvbd.2021.100028>. |
| 16. | Castro, O.; Venzal, J.M.; Félix, M.L. Two new records of helminth parasites of domestic cat from Uruguay: Alaria alata (Goeze, 1782) (Digenea, Diplostomidae) and Lagochilascaris major Leiper, 1910 (Nematoda, Ascarididae). *Vet Parasitol* **2009**, *160*, 344-347, doi:10.1016/j.vetpar.2008.11.019. |
| 17. | Cirak, V.Y.; Senlik, B.; Aydogdu, A.; Selver, M.; Akyol, V. Helminth parasites found in hedgehogs (Erinaceus concolor) from Turkey. *Prev Vet Med* **2010**, *97*, 64-66, doi:10.1016/j.prevetmed.2010.07.007. |
| 18. | Criado-Fornelio, A.; Gutierrez-Garcia, L.; Rodriguez-Caabeiro, F.; Reus-Garcia, E.; Roldan-Soriano, M.A.; Diaz-Sanchez, M.A. A parasitological survey of wild red foxes (Vulpes vulpes) from the province of Guadalajara, Spain. *Veterinary Parasitology* **2000**, *92*, 245-251, doi:<https://doi.org/10.1016/S0304-4017(00)00329-0>. |
| 19. | Crisi, P.E.; Aste, G.; Traversa, D.; Di Cesare, A.; Febo, E.; Vignoli, M.; Santori, D.; Luciani, A.; Boari, A. Single and mixed feline lungworm infections: clinical, radiographic and therapeutic features of 26 cases (2013-2015). *J Feline Med Surg* **2017**, *19*, 1017-1029, doi:10.1177/1098612x16670563. |
| 20. | Crisi, P.E.; Paoletti, B.; Morelli, S.; Simonato, G.; Colombo, M.; Tiscar, P.G.; Boari, A. Tritrichomonas foetus in cats from Central Italy: Clinical signs and risk factors. *Vet Parasitol Reg Stud Reports* **2021**, *24*, 100577, doi:10.1016/j.vprsr.2021.100577. |
| 21. | Crum, J.M.; Nettles, V.F.; Davidson, W.R. Studies on endoparasites of the black bear (Ursus americanus) in the southeastern United States. *J Wildl Dis* **1978**, *14*, 178-186, doi:10.7589/0090-3558-14.2.178. |
| 22. | Davidson, R.K.; Gjerde, B.; Vikøren, T.; Lillehaug, A.; Handeland, K. Prevalence of Trichinella larvae and extra-intestinal nematodes in Norwegian red foxes (Vulpes vulpes). *Vet Parasitol* **2006**, *136*, 307-316, doi:10.1016/j.vetpar.2005.11.015. |
| 23. | Deak, G.; Gherman, C.M.; Ionică, A.M.; Péter, Á.; Sándor, D.A.; Mihalca, A.D. Biotic and abiotic factors influencing the prevalence, intensity and distribution of Eucoleus aerophilus and Crenosoma vulpis in red foxes, Vulpes vulpes from Romania. *Int J Parasitol Parasites Wildl* **2020**, *12*, 121-125, doi:10.1016/j.ijppaw.2020.05.009. |
| 24. | Deak, G.; Ionica, A.M.; Pop, R.A.; Mihalca, A.D.; Gherman, C.M. New insights into the distribution of cardio-pulmonary nematodes in road-killed wild felids from Romania. *Parasites & Vectors* **2022**, *15*, doi:10.1186/s13071-022-05281-z. |
| 25. | Di Cesare, A.; Castagna, G.; Meloni, S.; Milillo, P.; Latrofa, S.; Otranto, D.; Traversa, D. Canine and feline infections by cardiopulmonary nematodes in central and southern Italy. *Parasitol Res* **2011**, *109 Suppl 1*, S87-96, doi:10.1007/s00436-011-2405-5. |
| 26. | Di Cesare, A.; Morelli, S.; Morganti, G.; Simonato, G.; Veronesi, F.; Colombo, M.; Berlanda, M.; Lebon, W.; Gallo, M.; Beugnet, F.; et al. Efficacy of milbemycin oxime/afoxolaner chewable tablets (NEXGARD SPECTRA(®)) against Capillaria aerophila and Capillaria boehmi in naturally infected dogs. *Parasit Vectors* **2021**, *14*, 143, doi:10.1186/s13071-021-04648-y. |
| 27. | Di Cesare, A.; Veronesi, F.; Grillotti, E.; Manzocchi, S.; Perrucci, S.; Beraldo, P.; Cazzin, S.; De Liberato, C.; Barros, L.A.; Simonato, G.; et al. Respiratory nematodes in cat populations of Italy. *Parasitol Res* **2015**, *114*, 4463-4469, doi:10.1007/s00436-015-4687-5. |
| 28. | Di Francesco, C.E.; Smoglica, C.; Paoletti, B.; Angelucci, S.; Innocenti, M.; Antonucci, A.; Di Domenico, G.; Marsilio, F. Detection of selected pathogens in Apennine wolf (Canis lupus italicus) by a non-invasive GPS-based telemetry sampling of two packs from Majella National Park, Italy. *Eur J Wildl Res* **2019**, *65*, 84, doi:10.1007/s10344-019-1326-y. |
| 29. | Diakou, A.; Migli, D.; Dimzas, D.; Morelli, S.; Di Cesare, A.; Youlatos, D.; Lymberakis, P.; Traversa, D. Endoparasites of European Wildcats (Felis silvestris) in Greece. *Pathogens* **2021**, *10*, doi:10.3390/pathogens10050594. |
| 30. | Estevez-Sanchez, E.; Checa, R.; Montoya, A.; Barrera, J.P.; Lopez-Beceiro, A.M.; Fidalgo, L.E.; Miro, G. A High Prevalence of Cardiopulmonary Worms Detected in the Iberian Wolf (Canis lupus): A Threat for Wild and Domestic Canids. *Animals* **2022**, *12*, doi:10.3390/ani12172289. |
| 31. | Falsone, L.; Brianti, E.; Gaglio, G.; Napoli, E.; Anile, S.; Mallia, E.; Giannelli, A.; Poglayen, G.; Giannetto, S.; Otranto, D. The European wildcats (Felis silvestris silvestris) as reservoir hosts of Troglostrongylus brevior (Strongylida: Crenosomatidae) lungworms. *Vet Parasitol* **2014**, *205*, 193-198, doi:10.1016/j.vetpar.2014.06.024. |
| 32. | Figueiredo, A.M.; Barros, T.; Valente, A.M.; Fonseca, C.; Carvalho, L.M.d.; Torres, R.T. Prevalence of zoonotic parasites in an endangered Iberian wolf (Canis lupus signatus) population in Portugal. *Mammalian Biology* **2019**, *98*, 154-162, doi:<https://doi.org/10.1016/j.mambio.2019.09.008>. |
| 33. | Fiorello, C.V.; Robbins, R.G.; Maffei, L.; Wade, S.E. Parasites of free-ranging small canids and felids in the Bolivian Chaco. *J Zoo Wildl Med* **2006**, *37*, 130-134, doi:10.1638/05-075.1. |
| 34. | Foster, S.F.; Martin, P.; Braddock, J.A.; Malik, R. A retrospective analysis of feline bronchoalveolar lavage cytology and microbiology (1995-2000). *J Feline Med Surg* **2004**, *6*, 189-198, doi:10.1016/j.jfms.2003.12.001. |
| 35. | Gaglio, G.; Allen, S.; Bowden, L.; Bryant, M.; Morgan, E.R. Parasites of European hedgehogs (Erinaceus europaeus) in Britain: epidemiological study and coprological test evaluation. *European Journal of Wildlife Research* **2010**, *56*, 839-844, doi:10.1007/s10344-010-0381-1. |
| 36. | Garrido-Castañé, I.; Ortuño, A.; Marco, I.; Castellà, J. Cardiopulmonary helminths in foxes from the Pyrenees. *Acta Parasitol* **2015**, *60*, 712-715, doi:10.1515/ap-2015-0101. |
| 37. | Gavrilović, P.; Dobrosavljević, I.; Vasković, N.; Todorović, I.; Živulj, A.; Kureljušić, B.; Pavlović, I. Cardiopulmonary parasitic nematodes of the red fox (Vulpes vulpes) in Serbia. *Acta Vet Hung* **2019**, *67*, 60-69, doi:10.1556/004.2019.007. |
| 38. | Giannelli, A.; Brianti, E.; Varcasia, A.; Colella, V.; Tamponi, C.; Di Paola, G.; Knaus, M.; Halos, L.; Beugnet, F.; Otranto, D. Efficacy of Broadline® spot-on against Aelurostrongylus abstrusus and Troglostrongylus brevior lungworms in naturally infected cats from Italy. *Veterinary Parasitology* **2015**, *209*, 273-277, doi:<https://doi.org/10.1016/j.vetpar.2015.02.037>. |
| 39. | Giannelli, A.; Capelli, G.; Joachim, A.; Hinney, B.; Losson, B.; Kirkova, Z.; René-Martellet, M.; Papadopoulos, E.; Farkas, R.; Napoli, E.; et al. Lungworms and gastrointestinal parasites of domestic cats: a European perspective. *Int J Parasitol* **2017**, *47*, 517-528, doi:10.1016/j.ijpara.2017.02.003. |
| 40. | Gillis-Germitsch, N.; Tritten, L.; Hegglin, D.; Deplazes, P.; Schnyder, M. Conquering Switzerland: the emergence of Angiostrongylus vasorum in foxes over three decades and its rapid regional increase in prevalence contrast with the stable occurrence of lungworms. *Parasitology* **2020**, *147*, 1071-1079, doi:10.1017/s0031182020000700. |
| 41. | Gortázar, C.; Villafuerte, R.; Lucientes, J.; Fernández-de-Luco, D. Habitat related differences in helminth parasites of red foxes in the Ebro valley. *Veterinary Parasitology* **1998**, *80*, 75-81, doi:<https://doi.org/10.1016/S0304-4017(98)00192-7>. |
| 42. | Guardone, L.; Magi, M.; Prati, M.C.; Macchioni, F. Cardiorespiratory and gastrointestinal parasites of dogs in north-west Italy. *Helminthologia* **2016**, *53*, 318-325, doi:10.1515/helmin-2016-0032. |
| 43. | Hansen, A.P.; Skarbye, L.K.; Vinther, L.M.; Willesen, J.L.; Pipper, C.B.; Olsen, C.S.; Mejer, H. Occurrence and clinical significance of Aelurostrongylus abstrusus and other endoparasites in Danish cats. *Vet Parasitol* **2017**, *234*, 31-39, doi:10.1016/j.vetpar.2016.12.015. |
| 44. | Hinney, B.; Gottwald, M.; Moser, J.; Reicher, B.; Schäfer, B.J.; Schaper, R.; Joachim, A.; Künzel, F. Examination of anonymous canine faecal samples provides data on endoparasite prevalence rates in dogs for comparative studies. *Veterinary Parasitology* **2017**, *245*, 106-115, doi:<https://doi.org/10.1016/j.vetpar.2017.08.016>. |
| 45. | Hodžić, A.; Alić, A.; Klebić, I.; Kadrić, M.; Brianti, E.; Duscher, G.G. Red fox (Vulpes vulpes) as a potential reservoir host of cardiorespiratory parasites in Bosnia and Herzegovina. *Vet Parasitol* **2016**, *223*, 63-70, doi:10.1016/j.vetpar.2016.04.016. |
| 46. | Hoggard, K.R.; Jarriel, D.M.; Bevelock, T.J.; Verocai, G.G. Prevalence survey of gastrointestinal and respiratory parasites of shelter cats in northeastern Georgia, USA. *Vet Parasitol Reg Stud Reports* **2019**, *16*, 100270, doi:10.1016/j.vprsr.2019.100270. |
| 47. | Holmes, P.R.; Kelly, J.D. Capillaria aerophila in the domestic cat in Australia. *Aust Vet J* **1973**, *49*, 472-473, doi:10.1111/j.1751-0813.1973.tb09296.x. |
| 48. | Hoopes, J.; Hill, J.E.; Polley, L.; Fernando, C.; Wagner, B.; Schurer, J.; Jenkins, E. Enteric parasites of free-roaming, owned, and rural cats in prairie regions of Canada. *Can Vet J* **2015**, *56*, 495-501. |
| 49. | Ilić, T.; Becskei, Z.; Petrović, T.; Polaček, V.; Ristić, B.; Milić, S.; Stepanović, P.; Radisavljević, K.; Dimitrijević, S. Endoparasitic fauna of red foxes (Vulpes vulpes) and golden jackals (Canis aureus) in Serbia. *Acta Parasitol* **2016**, *61*, 389-396, doi:10.1515/ap-2016-0051. |
| 50. | Ilic, T.; Becskei, Z.; Tasic, A.; Stepanovic, P.; Radisavljevic, K.; Duric, B.; Dimitrijevic, S. Red foxes (Vulpes vulpes) as reservoirs of respiratory capillariosis in Serbia. *Journal of Veterinary Research* **2016**, *60*, 153-157, doi:10.1515/jvetres-2016-0022. |
| 51. | Karamon, J.; Dąbrowska, J.; Kochanowski, M.; Samorek-Pieróg, M.; Sroka, J.; Różycki, M.; Bilska-Zając, E.; Zdybel, J.; Cencek, T. Prevalence of intestinal helminths of red foxes (Vulpes vulpes) in central Europe (Poland): a significant zoonotic threat. *Parasit Vectors* **2018**, *11*, 436, doi:10.1186/s13071-018-3021-3. |
| 52. | Kelly, D.J.; Marples, N.M.; Byrne, R.L.; Fogarty, U.; Kenny, K.; Cameron, H.; Griffin, D.; Holland, C.V. An investigation of Mycobacterium bovis and helminth coinfection in the European badger Meles meles. *Int J Parasitol Parasites Wildl* **2022**, *19*, 311-316, doi:10.1016/j.ijppaw.2022.11.001. |
| 53. | Knaus, M.; Kusi, I.; Rapti, D.; Xhaxhiu, D.; Winter, R.; Visser, M.; Rehbein, S. Endoparasites of cats from the Tirana area and the first report on Aelurostrongylus abstrusus (Railliet, 1898) in Albania. *Wien Klin Wochenschr* **2011**, *123 Suppl 1*, 31-35, doi:10.1007/s00508-011-1588-1. |
| 54. | Kotwa, J.D.; French, S.K.; Greer, T.; Elsemore, D.A.; Hanna, R.; Jardine, C.M.; Pearl, D.L.; Weese, J.S.; Mercer, N.; Peregrine, A.S. Prevalence of intestinal parasites in dogs in southern Ontario, Canada, based on fecal samples tested using sucrose double centrifugation and Fecal Dx® tests. *Veterinary Parasitology: Regional Studies and Reports* **2021**, *26*, 100618, doi:<https://doi.org/10.1016/j.vprsr.2021.100618>. |
| 55. | Krecek, R.C.; Moura, L.; Lucas, H.; Kelly, P. Parasites of stray cats (Felis domesticus L., 1758) on St. Kitts, West Indies. *Veterinary Parasitology* **2010**, *172*, 147-149, doi:<https://doi.org/10.1016/j.vetpar.2010.04.033>. |
| 56. | Krone, O.; Guminsky, O.; Meinig, H.; Herrmann, M.; Trinzen, M.; Wibbelt, G. Endoparasite spectrum of wild cats (Felis silvestris Schreber, 1777) and domestic cats (Felis catus L.) from the Eifel, Pfalz region and Saarland, Germany. *European Journal of Wildlife Research* **2008**, *54*, 95-100, doi:10.1007/s10344-007-0116-0. |
| 57. | Kurumadas, R.; Chennuru, S.; Krovvidi, S.; Pattipati, M. Assessing risk factors associated with prevalence of canine gastrointestinal parasitic zoonoses in Andhra Pradesh, India. *Indian Journal of Animal Sciences* **2020**, *90*, 851-855. |
| 58. | La Torre, F.; Di Cesare, A.; Simonato, G.; Cassini, R.; Traversa, D.; Frangipane di Regalbono, A. Prevalence of zoonotic helminths in Italian house dogs. *J Infect Dev Ctries* **2018**, *12*, 666-672, doi:10.3855/jidc.9865. |
| 59. | Lalošević, V.; Lalošević, D.; Capo, I.; Simin, V.; Galfi, A.; Traversa, D. High infection rate of zoonotic Eucoleus aerophilus infection in foxes from Serbia. *Parasite* **2013**, *20*, 3, doi:10.1051/parasite/2012003. |
| 60. | Lassnig, H.; Prosl, H.; Hinterdorfer, F. Parasites of the red fox (Vulpes vulpes) in Styria. *Wiener Tierarztliche Monatsschrift* **1998**, *85*, 116-122. |
| 61. | Laurimaa, L.; Moks, E.; Soe, E.; Valdmann, H.; Saarma, U. Echinococcus multilocularis and other zoonotic parasites in red foxes in Estonia. *Parasitology* **2016**, *143*, 1450-1458, doi:10.1017/s0031182016001013. |
| 62. | Laurimaa, L.; Suld, K.; Davison, J.; Moks, E.; Valdmann, H.; Saarma, U. Alien species and their zoonotic parasites in native and introduced ranges: The raccoon dog example. *Vet Parasitol* **2016**, *219*, 24-33, doi:10.1016/j.vetpar.2016.01.020. |
| 63. | Lemming, L.; Jørgensen, A.C.; Nielsen, L.B.; Nielsen, S.T.; Mejer, H.; Chriél, M.; Petersen, H.H. Cardiopulmonary nematodes of wild carnivores from Denmark: Do they serve as reservoir hosts for infections in domestic animals? *Int J Parasitol Parasites Wildl* **2020**, *13*, 90-97, doi:10.1016/j.ijppaw.2020.08.001. |
| 64. | Lopez, A.; Aburto, E.; Jones, K.; Robbins, W.; Conboy, G. EUCOLEUS BOEHMI INFECTION IN THE NASAL CONCHAE AND PARANASAL SINUSES OF RED FOX (VULPES VULPES) ON PRINCE EDWARD ISLAND, CANADA. *J Wildl Dis* **2016**, *52*, 279-285, doi:10.7589/2015-06-149. |
| 65. | Lucio-Forster, A.; Bowman, D.D. Prevalence of fecal-borne parasites detected by centrifugal flotation in feline samples from two shelters in upstate New York. *Journal of Feline Medicine & Surgery* **2011**, *13*, 300-303, doi:<https://doi.org/10.1016/j.jfms.2010.12.013>. |
| 66. | Magi, M.; Guardone, L.; Prati, M.C.; Mignone, W.; Macchioni, F. Extraintestinal nematodes of the red fox Vulpes vulpes in north-west Italy. *J Helminthol* **2015**, *89*, 506-511, doi:10.1017/s0022149x1400025x. |
| 67. | Magi, M.; Guardone, L.; Prati, M.C.; Torracca, B.; Macchioni, F. First report of Eucoleus boehmi (syn. Capillaria boehmi) in dogs in north-western Italy, with scanning electron microscopy of the eggs. *Parasite* **2012**, *19*, 433-435, doi:10.1051/parasite/2012194433. |
| 68. | Magi, M.; Macchioni, F.; Dell'omodarme, M.; Prati, M.C.; Calderini, P.; Gabrielli, S.; Iori, A.; Cancrini, G. Endoparasites of red fox (Vulpes vulpes) in central Italy. *J Wildl Dis* **2009**, *45*, 881-885, doi:10.7589/0090-3558-45.3.881. |
| 69. | Mañas, S.; Ferrer, D.; Castellà, J.; Maria López-Martı́n, J. Cardiopulmonary helminth parasites of red foxes (Vulpes vulpes) in Catalonia, northeastern Spain. *The Veterinary Journal* **2005**, *169*, 118-120, doi:<https://doi.org/10.1016/j.tvjl.2003.12.011>. |
| 70. | Manke, K.J.; Stoye, M. Parasitological studies of red foxes (Vulpes vulpes L.) in the northern districts of Schleswig-Holstein. *Tierarztliche Umschau* **1998**, *53*, 207-214. |
| 71. | Martinez-Carrasco, C.; de Ybanez, M.R.R.; Sagarminaga, J.L.; Garijo, M.M.; Moreno, F.; Acosta, I.; Hernandez, S.; Alonso, F.D. Parasites of the red fox (Vulpes vulpes Linnaeus, 1758) in Murcia, southeast Spain. *Revue De Medecine Veterinaire* **2007**, *158*, 331-335. |
| 72. | Martínez-Rondán, F.J.; Ruiz de Ybáñez, M.R.; López-Beceiro, A.M.; Fidalgo, L.E.; Berriatua, E.; Lahat, L.; Sacristán, I.; Oleaga, Á.; Martínez-Carrasco, C. Cardiopulmonary nematode infections in wild canids: Does the key lie on host-prey-parasite evolution? *Res Vet Sci* **2019**, *126*, 51-58, doi:10.1016/j.rvsc.2019.08.008. |
| 73. | Mircean, V.; Györke, A.; Cozma, V. Prevalence and risk factors of Giardia duodenalis in dogs from Romania. *Vet Parasitol* **2012**, *184*, 325-329, doi:10.1016/j.vetpar.2011.08.022. |
| 74. | Mircean, V.; Titilincu, A.; Vasile, C. Prevalence of endoparasites in household cat (Felis catus) populations from Transylvania (Romania) and association with risk factors. *Vet Parasitol* **2010**, *171*, 163-166, doi:10.1016/j.vetpar.2010.03.005. |
| 75. | Mizgajska-Wiktor, H.; Jarosz, W.; Piłacińska, B.; Dziemian, S. Helminths of hedgehogs, Erinaceus europaeus and E. roumanicus from Poznań region, Poland--coprological study. *Wiad Parazytol* **2010**, *56*, 329-332. |
| 76. | Morelli, S.; Colombo, M.; Traversa, D.; Iorio, R.; Paoletti, B.; Bartolini, R.; Barlaam, A.; Di Cesare, A. Zoonotic intestinal helminthes diagnosed in a 6-year period (2015–2020) in privately owned dogs of sub-urban and urban areas of Italy. *Veterinary Parasitology: Regional Studies and Reports* **2022**, *29*, 100689, doi:<https://doi.org/10.1016/j.vprsr.2022.100689>. |
| 77. | Morgan, E.R.; Tomlinson, A.; Hunter, S.; Nichols, T.; Roberts, E.; Fox, M.T.; Taylor, M.A. Angiostrongylus vasorum and Eucoleus aerophilus in foxes (Vulpes vulpes) in Great Britain. *Vet Parasitol* **2008**, *154*, 48-57, doi:10.1016/j.vetpar.2008.02.030. |
| 78. | Morrison, E.E.; Gier, H.T. Lungworms in coyotes on the Great Plains. *J Wildl Dis* **1978**, *14*, 314-316, doi:10.7589/0090-3558-14.3.314. |
| 79. | Morrison, E.E.; Gier, H.T. Parasitic infection of Filaroides osleri, Capillaria aerophila and Spirocera lupi in coyotes from the Southwestern United States. *J Wildl Dis* **1979**, *15*, 557-559, doi:10.7589/0090-3558-15.4.557. |
| 80. | Mugnaini, L.; Papini, R.; Gorini, G.; Passantino, A.; Merildi, V.; Mancianti, F. Pattern and predictive factors of endoparasitism in cats in Central Italy. *Revue De Medecine Veterinaire* **2012**, *163*, 89-94. |
| 81. | Naem, S.; Pourreza, B.; Gorgani-Firouzjaee, T. The European hedgehog (Erinaceus europaeus), as a reservoir for helminth parasites in Iran. *Vet Res Forum* **2015**, *6*, 149-153. |
| 82. | Nagamori, Y.; Payton, M.E.; Duncan-Decocq, R.; Johnson, E.M. Fecal survey of parasites in free-roaming cats in northcentral Oklahoma, United States. *Vet Parasitol Reg Stud Reports* **2018**, *14*, 50-53, doi:10.1016/j.vprsr.2018.08.008. |
| 83. | Nagamori, Y.; Payton, M.E.; Looper, E.; Apple, H.; Johnson, E.M. Retrospective survey of parasitism identified in feces of client-owned cats in North America from 2007 through 2018. *Vet Parasitol* **2020**, *277*, 109008, doi:10.1016/j.vetpar.2019.109008. |
| 84. | Napoli, E.; Anile, S.; Arrabito, C.; Scornavacca, D.; Mazzamuto, M.V.; Gaglio, G.; Otranto, D.; Giannetto, S.; Brianti, E. Survey on parasitic infections in wildcat (Felis silvestris silvestris Schreber, 1777) by scat collection. *Parasitol Res* **2016**, *115*, 255-261, doi:10.1007/s00436-015-4742-2. |
| 85. | Nevárez, A.; López, A.; Conboy, G.; Ireland, W.; Sims, D. Distribution of Crenosoma vulpis and Eucoleus aerophilus in the lung of free-ranging red foxes (Vulpes vulpes). *J Vet Diagn Invest* **2005**, *17*, 486-489, doi:10.1177/104063870501700516. |
| 86. | Nugaraitė, D.; Mažeika, V.; Paulauskas, A. Helminths of Mustelids with Overlapping Ecological Niches: Eurasian Otter Lutra Lutra (Linnaeus, 1758), American Mink Neovison Vison Schreber, 1777, and European Polecat Mustela Putorius Linnaeus, 1758. *Helminthologia* **2019**, *56*, 66-74, doi:10.2478/helm-2018-0035. |
| 87. | Palmer, C.S.; Thompson, R.C.A.; Traub, R.J.; Rees, R.; Robertson, I.D. National study of the gastrointestinal parasites of dogs and cats in Australia. *Veterinary Parasitology* **2008**, *151*, 181-190, doi:<https://doi.org/10.1016/j.vetpar.2007.10.015>. |
| 88. | Panayotova-Pancheva, M.; Dakova, V. New Data on Helminth Parasites of the Stone Marten Martes foina (Erxleben, 1777) (Carnivora: Mustelidae) in Bulgaria. *Acta Zoologica Bulgarica* **2021**, *73*, 113-118. |
| 89. | Paoletti, B.; Iorio, R.; Traversa, D.; Di Francesco, C.E.; Gentile, L.; Angelucci, S.; Amicucci, C.; Bartolini, R.; Marangi, M.; Di Cesare, A. Helminth infections in faecal samples of Apennine wolf (Canis lupus italicus) and Marsican brown bear (Ursus arctos marsicanus) in two protected national parks of central Italy. *Ann Parasitol* **2017**, *63*, 205-212, doi:10.17420/ap6303.107. |
| 90. | Paoletti, B.; Traversa, D.; Iorio, R.; De Berardinis, A.; Bartolini, R.; Salini, R.; Di Cesare, A. Zoonotic parasites in feces and fur of stray and private dogs from Italy. *Parasitol Res* **2015**, *114*, 2135-2141, doi:10.1007/s00436-015-4402-6. |
| 91. | Pipikova, J.; Papajova, I.; Soltys, J.; Schusterova, I.; Kocisova, D.; Tohathyova, A. Segregated settlements present an increased risk for the parasite infections spread in Northeastern Slovakia. *Helminthologia* **2017**, *54*, 199-210, doi:10.1515/helm-2017-0026. |
| 92. | Popiołek, M.; Szczesnaa, J.; Nowaka, S.; Mysłajeka, R.W. Helminth infections in faecal samples of wolves Canis lupus L. from the western Beskidy Mountains in southern Poland. *J Helminthol* **2007**, *81*, 339-344, doi:10.1017/s0022149x07821286. |
| 93. | Rajkovic-Janje, R.; Marinculic, A.; Bosnic, S.; Benic, M.; Vinkovic, B.; Mihaljevic, Z. Prevalence and seasonal distribution of helminth parasites in red foxes (Vulpes vulpes) from the Zagreb County (Croatia). *Zeitschrift Fur Jagdwissenschaft* **2002**, *48*, 151-160, doi:10.1007/bf02189989. |
| 94. | Raschka, C.; Haupt, W.; Ribbeck, R. STUDIES ON ENDOPARASITIZATION OF STRAY CAT. *Monatshefte Fur Veterinarmedizin* **1994**, *49*, 307-315. |
| 95. | Rasmussen, S.L.; Hallig, J.; van Wijk, R.E.; Petersen, H.H. An investigation of endoparasites and the determinants of parasite infection in European hedgehogs (Erinaceus europaeus) from Denmark. *Int J Parasitol Parasites Wildl* **2021**, *16*, 217-227, doi:10.1016/j.ijppaw.2021.10.005. |
| 96. | Rehbein, S.; Kaulfuß, K.H.; Visser, M.; Sommer, M.F.; Grimm, F.; Silaghi, C. Parasites of sheep herding dogs in central Germany. *Berl Munch Tierarztl Wochenschr* **2016**, *129*, 56-64. |
| 97. | Remesar, S.; Garcia-Dios, D.; Calabuig, N.; Prieto, A.; Diaz-Cao, J.M.; Lopez-Lorenzo, G.; Lopez, C.; Fernandez, G.; Morrondo, P.; Panadero, R.; et al. Cardiorespiratory nematodes and co-infections with gastrointestinal parasites in new arrivals at dog and cat shelters in north-western Spain. *Transboundary and Emerging Diseases* **2022**, *69*, E3141-E3153, doi:10.1111/tbed.14670. |
| 98. | Richardson, D.J.; Owen, W.B.; Snyder, D.E. Helminth parasites of the raccoon (Procyon lotor) from north-central Arkansas. *J Parasitol* **1992**, *78*, 163-166. |
| 99. | Riggio, F.; Mannella, R.; Ariti, G.; Perrucci, S. Intestinal and lung parasites in owned dogs and cats from central Italy. *Vet Parasitol* **2013**, *193*, 78-84, doi:10.1016/j.vetpar.2012.11.026. |
| 100. | Saeed, I.; Maddox-Hyttel, C.; Monrad, J.; Kapel, C.M. Helminths of red foxes (Vulpes vulpes) in Denmark. *Vet Parasitol* **2006**, *139*, 168-179, doi:10.1016/j.vetpar.2006.02.015. |
| 101. | Sarvi, S.; Daryani, A.; Sharif, M.; Rahimi, M.T.; Azami, D.; Marhaba, Z.; Ahmadpour, E.; Mizani, A. Domestic dog as a human health hazard in north of Iran. *J Parasit Dis* **2016**, *40*, 930-934, doi:10.1007/s12639-014-0608-2. |
| 102. | Sato, H.; Inaba, T.; Ihama, Y.; Kamiya, H. Parasitological survey on wild carnivora in north-western Tohoku, Japan. *J Vet Med Sci* **1999**, *61*, 1023-1026, doi:10.1292/jvms.61.1023. |
| 103. | Sauda, F.; Malandrucco, L.; Macrì, G.; Scarpulla, M.; De Liberato, C.; Terracciano, G.; Fichi, G.; Berrilli, F.; Perrucci, S. Leishmania infantum, Dirofilaria spp. and other endoparasite infections in kennel dogs in central Italy. *Parasite* **2018**, *25*, 2, doi:10.1051/parasite/2018001. |
| 104. | Scaramozzino, P.; Carvelli, A.; Iacoponi, F.; De Liberato, C. Endoparasites in household and shelter dogs from Central Italy. *Int J Vet Sci Med* **2018**, *6*, 45-47, doi:10.1016/j.ijvsm.2018.04.003. |
| 105. | Schug, K.; Krämer, F.; Schaper, R.; Hirzmann, J.; Failing, K.; Hermosilla, C.; Taubert, A. Prevalence survey on lungworm (Angiostrongylus vasorum, Crenosoma vulpis, Eucoleus aerophilus) infections of wild red foxes (Vulpes vulpes) in central Germany. *Parasit Vectors* **2018**, *11*, 85, doi:10.1186/s13071-018-2672-4. |
| 106. | Segovia, J.M.; Torres, J.; Miquel, J. Helminth parasites of the red fox (*Vulpes vulpes* L., 1758) in the Iberian Peninsula: an ecological study. *Acta Parasitol* **2004**, *49*, 67-79. |
| 107. | Segovia, J.M.; Torres, J.; Miquel, J.; Sospedra, E.; Guerrero, R.; Feliu, C. Analysis of helminth communities of the pine marten, Martes martes, in Spain: Mainland and insular data. *Acta Parasitologica* **2007**, *52*, 156-164, doi:10.2478/s11686-007-0012-5. |
| 108. | Seville, R.S.; Addison, E.M. Nongastrointestinal helminths in marten (Martes americana) from Ontario, Canada. *J Wildl Dis* **1995**, *31*, 529-533, doi:10.7589/0090-3558-31.4.529. |
| 109. | Shamaev, N.D.; Fedotova, A.Y.; Galiullina, A.V.; Mukminov, M.N.; Shuralev, E.A. Prevalence And Risk Factors Of Toxoplasma-Like And Intestinal Parasites In Cats From Urbanized Area Of Tatarstan, Russia. *Research Journal of Pharmaceutical Biological and Chemical Sciences* **2018**, *9*, 465-471. |
| 110. | Simonato, G.; Cassini, R.; Morelli, S.; Di Cesare, A.; La Torre, F.; Marcer, F.; Traversa, D.; Pietrobelli, M.; Frangipane di Regalbono, A. Contamination of Italian parks with canine helminth eggs and health risk perception of the public. *Preventive Veterinary Medicine* **2019**, *172*, 104788, doi:<https://doi.org/10.1016/j.prevetmed.2019.104788>. |
| 111. | Simonato, G.; Danesi, P.; Frangipane di Regalbono, A.; Dotto, G.; Tessarin, C.; Pietrobelli, M.; Pasotto, D. Surveillance of Zoonotic Parasites in Animals Involved in Animal-Assisted Interventions (AAIs). *Int J Environ Res Public Health* **2020**, *17*, doi:10.3390/ijerph17217914. |
| 112. | Skírnisson, K.; Eydal, M.; Gunnarsson, E.; Hersteinsson, P. Parasites of the arctic fox (Alopex lagopus) in Iceland. *J Wildl Dis* **1993**, *29*, 440-446, doi:10.7589/0090-3558-29.3.440. |
| 113. | Šmigová, J.; Papajová, I.; Šoltys, J.; Pipiková, J.; Šmiga, Ľ.; Šnábel, V.; Takáčová, J.; Takáč, L. The occurence of endoparasites in Slovakian household dogs and cats. *Vet Res Commun* **2021**, *45*, 243-249, doi:10.1007/s11259-021-09804-4. |
| 114. | Smith, H.J. Parasites of red foxes in New Brunswick and Nova Scotia. *J Wildl Dis* **1978**, *14*, 366-370, doi:10.7589/0090-3558-14.3.366. |
| 115. | Spada, E.; Proverbio, D.; Della Pepa, A.; Domenichini, G.; Bagnagatti De Giorgi, G.; Traldi, G.; Ferro, E. Prevalence of faecal-borne parasites in colony stray cats in northern Italy. *J Feline Med Surg* **2013**, *15*, 672-677, doi:10.1177/1098612x12473467. |
| 116. | Sréter, T.; Széll, Z.; Marucci, G.; Pozio, E.; Varga, I. Extraintestinal nematode infections of red foxes (Vulpes vulpes) in Hungary. *Vet Parasitol* **2003**, *115*, 329-334, doi:10.1016/s0304-4017(03)00217-6. |
| 117. | Steinbach, G.; Welzel, A.; Vonkeyserlingk, M.; Stoye, M. ON THE HELMINTHIC FAUNA OF THE RED FOX (VULPES-VULPES L) IN SOUTHERN LOWER SAXONY .1. NEMATODES AND TREMATODES. *Zeitschrift Fur Jagdwissenschaft* **1994**, *40*, 30-39, doi:10.1007/bf02241503. |
| 118. | Stepanović, P.; Despotović, D.; Dimitrijević, S.; Ilić, T. Clinical-parasitological Screening for Respiratory Capillariosis in Cats in Urban Environments. *Helminthologia* **2020**, *57*, 322-334, doi:10.2478/helm-2020-0046. |
| 119. | Stuart, P.; Golden, O.; Zintl, A.; de Waal, T.; Mulcahy, G.; McCarthy, E.; Lawton, C. A coprological survey of parasites of wild carnivores in Ireland. *Parasitol Res* **2013**, *112*, 3587-3593, doi:10.1007/s00436-013-3544-7. |
| 120. | Symeonidou, I.; Gelasakis, A.I.; Arsenopoulos, K.; Angelou, A.; Beugnet, F.; Papadopoulos, E. Feline gastrointestinal parasitism in Greece: emergent zoonotic species and associated risk factors. *Parasit Vectors* **2018**, *11*, 227, doi:10.1186/s13071-018-2812-x. |
| 121. | Szafrańska, E.; Wasielewski, O.; Bereszyński, A. A faecal analysis of helminth infections in wild and captive wolves, Canis lupus L., in Poland. *J Helminthol* **2010**, *84*, 415-419, doi:10.1017/s0022149x10000106. |
| 122. | Szczesna, J.; Popiołek, M.; Schmidt, K.; Kowalczyk, R. Coprological study on helminth fauna in Eurasian lynx (Lynx lynx) from the Białowieza Primeval Forest in eastern Poland. *J Parasitol* **2008**, *94*, 981-984, doi:10.1645/ge-1440.1. |
| 123. | Takács, A.; Szabó, L.; Juhász, L.; Takács, A.A.; Lanszki, J.; Takács, P.T.; Heltai, M. Data on the parasitological status of golden jackal (Canis aureus L., 1758) in Hungary. *Acta Vet Hung* **2014**, *62*, 33-41, doi:10.1556/AVet.2013.058. |
| 124. | Takacs, A.; Szemethy, L.; Heltai, M.; Takacs, A.A. Data on the parasitological state of wild cats (Felis silvestris Schreber 1777) and of their hybrids with feral domestic cats (Felis silvestris catus L. 1758) on Hungarian hunting areas. *Magyar Allatorvosok Lapja* **2011**, *133*, 670-674. |
| 125. | Takacs, A.; Szemethy, L.; Takacs, A.A.; Takacs, P.T.; Heltai, M. Data on the parasitological state of the Eurasian badger (Meles meles) in Hungary. *Magyar Allatorvosok Lapja* **2012**, *134*, 106-110. |
| 126. | Takeuchi-Storm, N.; Al-Sabi, M.N.S.; Chriel, M.; Enemark, H.L. Systematic examination of the cardiopulmonary, urogenital, muscular and gastrointestinal parasites of the Eurasian otters (Lutra lutra) in Denmark, a protected species recovering from a dramatic decline. *Parasitol Int* **2021**, *84*, 102418, doi:10.1016/j.parint.2021.102418. |
| 127. | Takeuchi-Storm, N.; Mejer, H.; Al-Sabi, M.N.; Olsen, C.S.; Thamsborg, S.M.; Enemark, H.L. Gastrointestinal parasites of cats in Denmark assessed by necropsy and concentration McMaster technique. *Vet Parasitol* **2015**, *214*, 327-332, doi:10.1016/j.vetpar.2015.06.033. |
| 128. | Tamponi, C.; Varcasia, A.; Pinna, S.; Melis, E.; Melosu, V.; Zidda, A.; Sanna, G.; Pipia, A.P.; Zedda, M.T.; Pau, S.; et al. Endoparasites detected in faecal samples from dogs and cats referred for routine clinical visit in Sardinia, Italy. *Veterinary Parasitology: Regional Studies and Reports* **2017**, *10*, 13-17, doi:<https://doi.org/10.1016/j.vprsr.2017.07.001>. |
| 129. | Taylor, C.S.; Garcia Gato, R.; Learmount, J.; Aziz, N.A.; Montgomery, C.; Rose, H.; Coulthwaite, C.L.; McGarry, J.W.; Forman, D.W.; Allen, S.; et al. Increased prevalence and geographic spread of the cardiopulmonary nematode Angiostrongylus vasorum in fox populations in Great Britain. *Parasitology* **2015**, *142*, 1190-1195, doi:10.1017/s0031182015000463. |
| 130. | Thiess, A.; Schuster, R.; Nöckler, K.; Mix, H. [Helminth findings in indigenous raccoon dogs Nyctereutes procyonoides (Gray, 1843)]. *Berl Munch Tierarztl Wochenschr* **2001**, *114*, 273-276. |
| 131. | Tiekotter, K.L. Helminth species diversity and biology in the bobcat, Lynx rufus (Schreber), from Nebraska. *J Parasitol* **1985**, *71*, 227-234. |
| 132. | Tolnai, Z.; Széll, Z.; Sréter, T. Environmental determinants of the spatial distribution of Angiostrongylus vasorum, Crenosoma vulpis and Eucoleus aerophilus in Hungary. *Vet Parasitol* **2015**, *207*, 355-358, doi:10.1016/j.vetpar.2014.12.008. |
| 133. | Torres, J.; Garciá-Perea, R.; Gisbert, J.; Feliu, C. Helminth fauna of the Iberian lynx, Lynx pardinus. *J Helminthol* **1998**, *72*, 221-226, doi:10.1017/s0022149x00016473. |
| 134. | Torres, J.; Miquel, J.; Fournier, P.; Fournier-Chambrillon, C.; Liberge, M.; Fons, R.; Feliu, C. Helminth communities of the autochthonous mustelids Mustela lutreola and M. putorius and the introduced Mustela vison in south-western France. *J Helminthol* **2008**, *82*, 349-355, doi:10.1017/s0022149x08046920. |
| 135. | Traversa, D.; Cesare, A.D.; Milillo, P.; Iorio, R.; Otranto, D. Infection by Eucoleus aerophilus in dogs and cats: Is another extra-intestinal parasitic nematode of pets emerging in Italy? *Research in Veterinary Science* **2009**, *87*, 270-272, doi:<https://doi.org/10.1016/j.rvsc.2009.02.006>. |
| 136. | Traversa, D.; Di Cesare, A.; Di Giulio, E.; Castagna, G.; Schaper, R.; Braun, G.; Lohr, B.; Pampurini, F.; Milillo, P.; Strube, K. Efficacy and safety of imidacloprid 10%/moxidectin 1% spot-on formulation in the treatment of feline infection by Capillaria aerophila. *Parasitol Res* **2012**, *111*, 1793-1798, doi:10.1007/s00436-012-3025-4. |
| 137. | Traversa, D.; Di Cesare, A.; Simonato, G.; Cassini, R.; Merola, C.; Diakou, A.; Halos, L.; Beugnet, F.; di Regalbono, A.F. Zoonotic intestinal parasites and vector-borne pathogens in Italian shelter and kennel dogs. *Comparative Immunology Microbiology and Infectious Diseases* **2017**, *51*, 69-75, doi:10.1016/j.cimid.2017.04.003. |
| 138. | Traversa, D.; Morelli, S.; Cassini, R.; Crisi, P.E.; Russi, I.; Grillotti, E.; Manzocchi, S.; Simonato, G.; Beraldo, P.; Viglietti, A.; et al. Occurrence of canine and feline extra-intestinal nematodes in key endemic regions of Italy. *Acta Trop* **2019**, *193*, 227-235, doi:10.1016/j.actatropica.2019.03.009. |
| 139. | Tull, A.; Moks, E.; Saarma, U. Endoparasite prevalence and infection risk factors among cats in an animal shelter in Estonia. *Folia Parasitol (Praha)* **2021**, *68*, doi:10.14411/fp.2021.010. |
| 140. | Veronesi, F.; Traversa, D.; Lepri, E.; Morganti, G.; Vercillo, F.; Grelli, D.; Cassini, R.; Marangi, M.; Iorio, R.; Ragni, B.; et al. OCCURRENCE OF LUNGWORMS IN EUROPEAN WILDCATS (FELIS SILVESTRIS SILVESTRIS) OF CENTRAL ITALY. *J Wildl Dis* **2016**, *52*, 270-278, doi:10.7589/2015-07-187. |
| 141. | Wierzbowska, I.A.; Kornaś, S.; Piontek, A.M.; Rola, K. The Prevalence of Endoparasites of Free Ranging Cats (Felis catus) from Urban Habitats in Southern Poland. *Animals (Basel)* **2020**, *10*, doi:10.3390/ani10040748. |
