# Supplementary material for: Occurrence of Eucoleus aerophilus in wild and domestic animals: a systematic review and meta-analysis
Source: Parasit Vectors. 2023 Jul 20;16:245. doi: 10.1186/s13071-023-05830-0 (PMC10360280; doi:10.1186/s13071-023-05830-0)
Supplement: Supplementary file 2 — Additional file 2: Table S2. List of excluded studies. [file 13071_2023_5830_MOESM2_ESM.docx]

**Tabel S2**. List of excluded studies.

| NO. | REFERENCES | Reason for exclusion |
| --- | --- | --- |
| 1. | Acosta, L.; León-Quinto, T.; Bornay-Llinares, F.J.; Simón, M.A.; Esteban, J.G. Helminth parasites in faecal samples from the endangered Iberian lynx (Lynx pardinus). Veterinary Parasitology 2011, 179, 175-179, doi:https://doi.org/10.1016/j.vetpar.2011.01.058. | Not a primary research |
| 2. | Arcenillas-Hernandez, I.; de Ybanez, M.R.R.; Tizzani, P.; Perez-Cutillas, P.; Martinez-Carrasco, C. Pearsonema plica in red foxes (Vulpes vulpes) from semi-arid areas of the Iberian Peninsula. International Journal for Parasitology-Parasites and Wildlife 2022, 19, 78-83, doi:10.1016/j.ijppaw.2022.08.005. | Not concerning information about *Eucoleus aerophilus* |
| 3. | Balestrieri, A.; Remonti, L.; Ferrari, N.; Ferrari, A.; Lo Valvo, T.; Robetto, S.; Orusa, R. Sarcoptic mange in wild carnivores and its co-occurrence with parasitic helminths in the Western Italian Alps. European Journal of Wildlife Research 2006, 52, 196-201, doi:10.1007/s10344-006-0036-4. | No epidemilogical/prevalence data |
| 4. | Barrs, V.R.; Martin, P.; Nicoll, R.G.; Beatty, J.A.; Malik, R. Pulmonary cryptococcosis and Capillaria aerophila infection in an FIV-positive cat. Aust Vet J 2000, 78, 154-158, doi:10.1111/j.1751-0813.2000.tb10581.x. | Case report |
| 5. | Beck, W. [Endoparasites of the hedgehog]. Wien Klin Wochenschr 2007, 119, 40-44, doi:10.1007/s00508-007-0860-x. | Not concerning information about *Eucoleus aerophilus* |
| 6. | Bisterfeld, K.; Raulf, M.K.; Waindok, P.; Springer, A.; Lang, J.; Lierz, M.; Siebert, U.; Strube, C. Cardio-pulmonary parasites of the European wildcat (Felis silvestris) in Germany. Parasit Vectors 2022, 15, 452, doi:10.1186/s13071-022-05578-z. | No species affiliation |
| 7. | Blagburn, B.L.; Swango, L.J.; Hendrix, C.M.; Lindsay, D.S. Comparative efficacies of ivermectin, febantel, fenbendazole, and mebendazole against helminth parasites of gray foxes. J Am Vet Med Assoc 1986, 189, 1084-1085. | No epidemilogical/prevalence data |
| 8. | Boussarie, D. Consultation of the European hedgehog. Point Veterinaire 2006, 37, 26-+. | No epidemilogical/prevalence data |
| 9. | Burgess, H.; Ruotsalo, K.; Peregrine, A.S.; Hanselman, B.; Abrams-Ogg, A. Eucoleus aerophilus respiratory infection in a dog with Addison's disease. Can Vet J 2008, 49, 389-392. | Case report |
| 10. | Conboy, G. Helminth Parasites of the Canine and Feline Respiratory Tract. Veterinary Clinics of North America-Small Animal Practice 2009, 39, 1109-+, doi:10.1016/j.cvsm.2009.06.006. | Review article |
| 11. | Corwin, R.M.; Pratt, S.E.; McCurdy, H.D. Anthelmintic effect of febantel/praziquantel paste in dogs and cats. Am J Vet Res 1984, 45, 154-155. | No epidemilogical/prevalence data |
| 12. | Cox, D.D.; Mullee, M.T. The fox lungworm (Capillaria aerophila) in a native cat in Eastern Kansas. Vet Med Small Anim Clin 1967, 62, 969-971. | Case report |
| 13. | Di Cesare, A.; Castagna, G.; Meloni, S.; Otranto, D.; Traversa, D. Mixed trichuroid infestation in a dog from Italy. Parasit Vectors 2012, 5, 128, doi:10.1186/1756-3305-5-128. | Case report |
| 14. | Di Cesare, A.; Castagna, G.; Otranto, D.; Meloni, S.; Milillo, P.; Latrofa, M.S.; Paoletti, B.; Bartolini, R.; Traversa, D. Molecular detection of Capillaria aerophila, an agent of canine and feline pulmonary capillariosis. J Clin Microbiol 2012, 50, 1958-1963, doi:10.1128/jcm.00103-12. | No epidemilogical/prevalence data |
| 15. | Di Cesare, A.; Iorio, R.; Crisi, P.; Paoletti, B.; Di Costanzo, R.; Dimitri, C.F.; Traversa, D. Treatment of Troglostrongylus brevior (Metastrongyloidea, Crenosomatidae) in mixed lungworm infections using spot-on emodepside. J Feline Med Surg 2015, 17, 181-185, doi:10.1177/1098612x14533552. | Case report |
| 16. | Di Cesare, A.; Otranto, D.; Latrofa, M.S.; Veronesi, F.; Perrucci, S.; Lalosevic, D.; Gherman, C.M.; Traversa, D. Genetic variability of Eucoleus aerophilus from domestic and wild hosts. Res Vet Sci 2014, 96, 512-515, doi:10.1016/j.rvsc.2014.03.018. | No epidemilogical/prevalence data |
| 17. | Di Cesare, A.; Veronesi, F.; Capelli, G.; Deuster, K.; Schaper, R.; Basano, F.S.; Nazzari, R.; Paoletti, B.; Traversa, D. Evaluation of the Efficacy and Safety of an Imidacloprid 10 %/Moxidectin 1 % Spot-on Formulation (Advocate (R), Advantage (R) Multi) in Cats Naturally Infected with Capillaria aerophila. Parasitology Research 2017, 116, S55-S64, doi:10.1007/s00436-017-5491-1. | No epidemilogical/prevalence data |
| 18. | Di Cesare, A.; Veronesi, F.; Frangipane di Regalbono, A.; De Liberato, C.; Perrucci, S.; Iorio, R.; Morganti, G.; Marangi, M.; Simonato, G.; Traversa, D. PCR-based assay for the mitochondrial cox1 specific amplification of Eucoleus böhmi. Veterinary Parasitology 2015, 211, 67-70, doi:https://doi.org/10.1016/j.vetpar.2015.04.016. | Not concerning information about *Eucoleus aerophilus* |
| 19. | Diakou, A.; Dimzas, D.; Astaras, C.; Savvas, I.; Di Cesare, A.; Morelli, S.; Neofitos, Κ.; Migli, D.; Traversa, D. Clinical investigations and treatment outcome in a European wildcat (Felis silvestris silvestris) infected by cardio-pulmonary nematodes. Vet Parasitol Reg Stud Reports 2020, 19, 100357, doi:10.1016/j.vprsr.2019.100357. | Case report |
| 20. | Elhamiani Khatat, S.; Rosenberg, D.; Benchekroun, G.; Polack, B. Lungworm Eucoleus aerophilus (Capillaria aerophila) infection in a feline immunodeficiency virus-positive cat in France. JFMS Open Rep 2016, 2, 2055116916651649, doi:10.1177/2055116916651649. | Case report |
| 21. | Evinger, J.V.; Kazacos, K.R.; Cantwell, H.D. Ivermectin for treatment of nasal capillariasis in a dog. J Am Vet Med Assoc 1985, 186, 174-175. | Not concerning information about *Eucoleus aerophilus* |
| 22. | Febo, E.; Crisi, P.E.; Traversa, D.; Luciani, A.; Di Tommaso, M.; Pantaleo, S.; Santori, D.; Di Cesare, A.; Boari, A.; Terragni, R.; et al. Comparison of clinical and imaging findings in cats with single and mixed lungworm infection. J Feline Med Surg 2019, 21, 581-589, doi:10.1177/1098612x18793445. | Review article |
| 23. | Foster, S.F.; Martin, P.; Allan, G.S.; Barrs, V.R.; Malik, R. Lower respiratory tract infections in cats: 21 cases (1995-2000). J Feline Med Surg 2004, 6, 167-180, doi:10.1016/j.jfms.2003.11.006. | Not a primary research |
| 24. | French, A.F.; Castillo-Alcala, F.; Gedye, K.R.; Knox, M.A.; Roe, W.D.; Gartrell, B.D. Ventral dermatitis in rowi (Apteryx rowi) caused by cutaneous capillariasis. International Journal for Parasitology: Parasites and Wildlife 2020, 13, 160-170, doi:https://doi.org/10.1016/j.ijppaw.2020.10.003. | Not concerning information about *Eucoleus aerophilus* |
| 25. | Guardone, L.; Deplazes, P.; Macchioni, F.; Magi, M.; Mathis, A. Ribosomal and mitochondrial DNA analysis of Trichuridae nematodes of carnivores and small mammals. Vet Parasitol 2013, 197, 364-369, doi:10.1016/j.vetpar.2013.06.022. | No epidemilogical/prevalence data |
| 26. | Guardone, L.; Schnyder, M.; Macchioni, F.; Deplazes, P.; Magi, M. Serological detection of circulating Angiostrongylus vasorum antigen and specific antibodies in dogs from central and northern Italy. Vet Parasitol 2013, 192, 192-198, doi:10.1016/j.vetpar.2012.10.016. | No epidemilogical/prevalence data |
| 27. | Henke, S.E.; Pence, D.B.; Bryant, F.C. Effect of short-term coyote removal on populations of coyote helminths. J Wildl Dis 2002, 38, 54-67, doi:10.7589/0090-3558-38.1.54. | No epidemilogical/prevalence data |
| 28. | Herman, L.H. Capillaria aerophila infection in a cat. Vet Med Small Anim Clin 1967, 62, 466-468. | Case report |
| 29. | Hermosilla, C.; Kleinertz, S.; Silva, L.M.R.; Hirzmann, J.; Huber, D.; Kusak, J.; Taubert, A. Protozoan and helminth parasite fauna of free-living Croatian wild wolves (Canis lupus) analyzed by scat collection. Veterinary Parasitology 2017, 233, 14-19, doi:https://doi.org/10.1016/j.vetpar.2016.11.011. | No species affiliation |
| 30. | Hernandez, J. Diagnostic des affections respiratoires d’origine parasitaire chez le chien et le chat. Pratique Médicale et Chirurgicale de l'Animal de Compagnie 2011, 46, 17-22, doi:https://doi.org/10.1016/j.anicom.2010.11.002. | Not concerning information about *Eucoleus aerophilus* |
| 31. | Jitsamai, W.; Kesdangsakonwut, S.; Srirat, T.; Taweethavonsawat, P. Case Report: Molecular and Pathological Investigations of Zoonotic Anatrichosoma Spp.-Induced Ulcerative Pododermatitis in a Domestic Cat in Thailand. Front Vet Sci 2021, 8, 759814, doi:10.3389/fvets.2021.759814. | Not concerning information about *Eucoleus aerophilus* |
| 32. | Joekel, D.E.; Maier, S.; Huggel, K.; Schaper, R.; Deplazes, P. Specific Antibody Detection in Dogs with Filarial Infections. Parasitology Research 2017, 116, S81-S89, doi:10.1007/s00436-017-5494-y. | No epidemilogical/prevalence data |
| 33. | Kistler, W.M.; Brown, J.D.; Allison, A.B.; Nemeth, N.M.; Yabsley, M.J. First report of Angiostrongylus vasorum and Hepatozoon from a red fox (Vulpes vulpes) from West Virginia, USA. Vet Parasitol 2014, 200, 216-220, doi:10.1016/j.vetpar.2013.12.007. | Case report |
| 34. | Knaus, M.; Shukullari, E.; Rapti, D.; Rehbein, S. Efficacy of Broadline against Capillaria aerophila lungworm infection in cats. Parasitol Res 2015, 114, 1971-1975, doi:10.1007/s00436-015-4386-2. | No epidemilogical/prevalence data |
| 35. | Koller, B.; Hegglin, D.; Schnyder, M. A grid-cell based fecal sampling scheme reveals: land-use and altitude affect prevalence rates of Angiostrongylus vasorum and other parasites of red foxes (Vulpes vulpes). Parasitology Research 2019, 118, 2235-2245, doi:10.1007/s00436-019-06325-7. | No species affiliation |
| 36. | Lalosević, D.; Lalosević, V.; Klem, I.; Stanojev-Jovanović, D.; Pozio, E. Pulmonary capillariasis miming bronchial carcinoma. Am J Trop Med Hyg 2008, 78, 14-16. | Data on human |
| 37. | Little, S.E.; Barrett, A.W.; Beall, M.J.; Bowman, D.D.; Dangoudoubiyam, S.; Elsemore, D.A.; Liotta, J.; Lucio-Forster, A.; McCrann, D.J.; Snowden, K.F.; et al. Coproantigen Detection Augments Diagnosis of Common Nematode Infections in Dogs. Top Companion Anim Med 2019, 35, 42-46, doi:10.1053/j.tcam.2019.04.001. | No epidemilogical/prevalence data |
| 38. | Majeed, S.K.; Cooper, J.E. Lesions associated with a Capillaria infestation in the European hedgehog (Erinaceus europaeus). Journal of Comparative Pathology 1984, 94, 625-628, doi:https://doi.org/10.1016/0021-9975(84)90069-0. | No species affiliation |
| 39. | Majeed, S.K.; Morris, P.A.; Cooper, J.E. Occurrence of the lungworms Capillaria and Crenosoma spp. in British hedgehogs (Erinaceus europaeus). Journal of Comparative Pathology 1989, 100, 27-36, doi:https://doi.org/10.1016/0021-9975(89)90087-X. | No species affiliation |
| 40. | Manzocchi, S.; Venco, L.; Di Cesare, A. What is your diagnosis? Squash preparation from the lung of a hedgehog. Vet Clin Pathol 2016, 45, 715-716, doi:10.1111/vcp.12409. | Case report |
| 41. | Morandi, B.; Sabetti, M.C.; Veronesi, F.; Morganti, G.; Pietra, M.; Poglayen, G.; Linta, N.; Conboy, G.; Galuppi, R. A case of a dog refractory to different treatments for pulmonary capillariasis. Parasitol Res 2021, 120, 1137-1141, doi:10.1007/s00436-020-06940-9. | Case report |
| 42. | Morelli, S.; Diakou, A.; Colombo, M.; Di Cesare, A.; Barlaam, A.; Dimzas, D.; Traversa, D. Cat Respiratory Nematodes: Current Knowledge, Novel Data and Warranted Studies on Clinical Features, Treatment and Control. Pathogens 2021, 10, doi:10.3390/pathogens10040454. | Review article |
| 43. | Morelli, S.; Marruchella, G.; Passarelli, A.; Diakou, A.; Di Cesare, A.; Colombo, M.; di Regalbono, A.F.; Frate, A.; Traversa, D. An Unusual Case of Mixed Respiratory Capillariosis in a Dog. Pathogens 2021, 10, doi:10.3390/pathogens10020117. | Case report |
| 44. | Myšková, E.; Brož, M.; Fuglei, E.; Kvičerová, J.; Mácová, A.; Sak, B.; Kváč, M.; Ditrich, O. Gastrointestinal parasites of arctic foxes (Vulpes lagopus) and sibling voles (Microtus levis) in Spitsbergen, Svalbard. Parasitol Res 2019, 118, 3409-3418, doi:10.1007/s00436-019-06502-8. | No species affiliation |
| 45. | Nettles, V.F.; Prestwood, A.K.; Davidson, W.R. Severe parasitism in an opossum. J Wildl Dis 1975, 11, 419-420, doi:10.7589/0090-3558-11.3.419. | Case report |
| 46. | Nevarez-Garza, A.M.; Lopez, A.; Conboy, G.; Ireland, W. Hyperplastic and Metaplastic Changes in the Bronchi and Bronchioles of Red Foxes (Vulpes vulpes) Naturally Infected with Crenosoma vulpis and Eucoleus aerophilus. Journal of Animal and Veterinary Advances 2008, 7, 1252-1256. | Not a primary research |
| 47. | Nolan, T.J.; Smith, G. Time series analysis of the prevalence of endoparasitic infections in cats and dogs presented to a veterinary teaching hospital. Veterinary Parasitology 1995, 59, 87-96, doi:https://doi.org/10.1016/0304-4017(94)00742-U. | No epidemilogical/prevalence data |
| 48. | Osullivan, E.N. Helminth infections in owned and stray dogs in Co Cork, Ireland. Irish Veterinary Journal 1997, 50, 108-110. | Lack of full text |
| 49. | Pohly, A.G.; Nijveldt, E.A.; Stone, M.S.; Walden, H.D.S.; Ossiboff, R.J.; Conrado, F.O. Infection with the fox lungworm (Crenosoma vulpis) in two dogs from New England-Two clinical reports and updated geographic distribution in North America. Veterinary Parasitology- Regional Studies and Reports 2022, 30, doi:10.1016/j.vprsr.2022.100714. | Case report |
| 50. | Rehbein, S.; Capári, B.; Duscher, G.; Keidane, D.; Kirkova, Z.; Petkevičius, S.; Rapti, D.; Wagner, A.; Wagner, T.; Chester, S.T.; et al. Efficacy against nematode and cestode infections and safety of a novel topical fipronil, (S)-methoprene, eprinomectin and praziquantel combination product in domestic cats under field conditions in Europe. Veterinary Parasitology 2014, 202, 10-17, doi:https://doi.org/10.1016/j.vetpar.2014.02.032. | No epidemilogical/prevalence data |
| 51. | Ribas, A.; Milazzo, C.; Foronda, P.; Casanova, J.C. New data on helminths of stone marten, Martes foina (Carnivora, Mustelidae), in Italy. Helminthologia 2004, 41, 59-61. | Case report |
| 52. | Richards, D.T.; Harris, S.; Lewis, J.W. Epidemiological studies on intestinal helminth parasites of rural and urban red foxes (Vulpes vulpes) in the United Kingdom. Vet Parasitol 1995, 59, 39-51, doi:10.1016/0304-4017(94)00736-v. | No epidemilogical/prevalence data |
| 53. | Romashov, B.V. Three Capillariid species (Nematoda, Capillariidae) of carnivores (Carnivora) and discussion of system and evolution of the nematode family Capillariidae. 1. Redescription of Eucoleus aerophilus and E-boehmi. Zoologichesky Zhurnal 2000, 79, 1379-1391. | Lack of full text |
| 54. | Schnyder, M.; Stebler, K.; Naucke, T.J.; Lorentz, S.; Deplazes, P. Evaluation of a rapid device for serological in-clinic diagnosis of canine angiostrongylosis. Parasit Vectors 2014, 7, 72, doi:10.1186/1756-3305-7-72. | No epidemilogical/prevalence data |
| 55. | Schoning, P.; Dryden, M.W.; Gabbert, N.H. Identification of a nasal nematode (Eucoleus boehmi) in greyhounds. Vet Res Commun 1993, 17, 277-281, doi:10.1007/bf01839218. | No epidemilogical/prevalence data |
| 56. | Schulz, H.P. 3 FREQUENTLY OVERLOOKED FELINE INFECTIONS FROM NEMATODES OF THE SPECIES AELUROSTRONGYLUS-ABSTRUSUS, CAPILLARIA-AEROPHILA, AND OLLULANUS-TRICUSPIS. Zeitschrift Fur Versuchstierkunde 1981, 23, 186-186. | No epidemilogical/prevalence data |
| 57. | Schuster, R.; Heidecke, D.; Schierhorn, K. [Contributions to the parasite fauna of local hosts. 10. On the endoparasitic fauna of Felis silvestris]. Appl Parasitol 1993, 34, 113-120. | No species affiliation |
| 58. | Spriggs, M.C.; Kaloustian, L.L.; Gerhold, R.W. Endoparasites of American marten (Martes americana): Review of the literature and parasite survey of reintroduced American marten in Michigan. International Journal for Parasitology: Parasites and Wildlife 2016, 5, 240-248, doi:https://doi.org/10.1016/j.ijppaw.2016.07.001. | Review article |
| 59. | Stevanović, O.; Vujanić, D.; Dobrijević, M.; Nedić, D. Clinical Case of Respiratory Eucoleosis in a Dog from Bosnia and Herzegovina. Acta Parasitol 2019, 64, 218-221, doi:10.2478/s11686-018-00024-6. | Case report |
| 60. | Traversa, D.; Di Cesare, A.; Lia, R.P.; Castagna, G.; Meloni, S.; Heine, J.; Strube, K.; Milillo, P.; Otranto, D.; Meckes, O.; et al. New insights into morphological and biological features of Capillaria aerophila (Trichocephalida, Trichuridae). Parasitol Res 2011, 109 Suppl 1, S97-104, doi:10.1007/s00436-011-2406-4. | No epidemilogical/prevalence data |
| 61. | Traversa, D.; Cesare, A.D.; Milillo, P.; Iorio, R.; Otranto, D. Infection by Eucoleus aerophilus in dogs and cats: Is another extra-intestinal parasitic nematode of pets emerging in Italy? Research in Veterinary Science 2009, 87, 270-272, doi:https://doi.org/10.1016/j.rvsc.2009.02.006. | Duplicated |
| 62. | Varcasia, A.; Brianti, E.; Tamponi, C.; Pipia, A.P.; Cabras, P.A.; Mereu, M.; Dantas-Torres, F.; Scala, A.; Otranto, D. Simultaneous infection by four feline lungworm species and implications for the diagnosis. Parasitol Res 2015, 114, 317-321, doi:10.1007/s00436-014-4207-z. | Case report |
| 63. | Visser, M.; Messner, C.; Rehbein, S. Massive infestation with fur mites (Lynxacarus mustelae) of a stone marten (Martes foina) from Tyrol. Wien Klin Wochenschr 2011, 123 Suppl 1, 36-42, doi:10.1007/s00508-011-0005-0. | Case report |
| 64. | Waap, H.; Gomes, J.; Nunes, T. Parasite communities in stray cat populations from Lisbon, Portugal. J Helminthol 2014, 88, 389-395, doi:10.1017/s0022149x1300031x. | Not concerning information about *Eucoleus aerophilus* |
| 65. | Wysmolek, M.E.; Klockiewicz, M.; Dlugosz, E.; Wisniewski, M. Canine antibody response against Dirofilaria repens in natural occult and microfilaremic infections. Comp Immunol Microbiol Infect Dis 2022, 86, 101818, doi:10.1016/j.cimid.2022.101818. | No epidemilogical/prevalence data |
| 66. | Yasuda, N., Akuzawa, M., Maruyama, H., Izawa, M., Doi, T., 1993. Helminths of the Tsushima leopard cat (Felis bengalensis euptilura). J Wildl Dis 29, 153-155. | Case report |
| 67. | Yasuda, N., Ezaki, K., Akuzawa, M., Izawa, M., Doi, T., Sakaguchi, N., Tatara, M., 1994. Helminth survey of wildcats in Japan. J Vet Med Sci 56, 1069-1073. | Case report |
